# Low-Energy Isomers of the Magic Number H^+^(H_2_O)_21_ Cluster

**DOI:** 10.1021/acs.jpca.5c01977

**Published:** 2025-05-21

**Authors:** T.-H. Choi, E. V. Henderson, K. D. Jordan

**Affiliations:** Department of Chemistry, University of Pittsburgh, Pittsburgh, Pennsylvania 15260, United States

## Abstract

Electronic structure calculations are used to characterize low-energy isomers of H^+^(H_2_O)_21_. Eleven different classes of isomers, based on the (H_2_O)_20_ pentagonal dodecahedron with the excess proton localized on the surface (as a hydrated hydronium ion) and the “extra” water molecule located in the interior of the cluster, are characterized. In 10 of these classes, the internal water molecule is engaged in six 5-membered rings, but in the remaining class, which is predicted to start at only 0.6 kcal/mol above the global minimum, the internal water is engaged in a 4-membered ring, an additional 6-membered ring, and four 5-membered rings. In addition, isomers with two 4-membered rings and two 6-membered rings on the cluster surface are predicted to start at only ∼1.3 kcal/mol above the lowest-energy dodecahedral-based structure.

## Graphical abstract

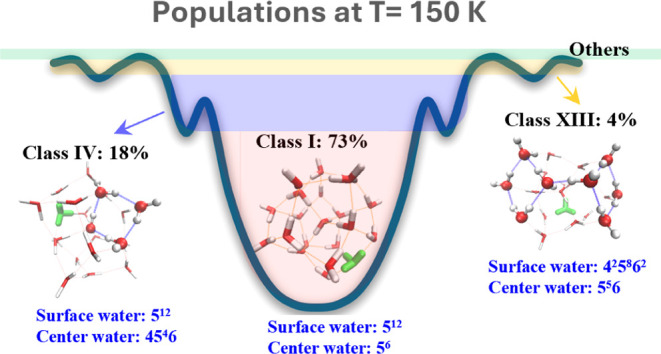

## 1 Introduction

As first reported by Searcy and Fenn,^2^ mass spectra of protonated water clusters reveal that the H^+^(H_2_O)*_n_*, *n* = 21, species is a magic number cluster, meaning that it is more stable per monomer than the neighboring *n* = 20 and 22 clusters. Although there was some speculation in the early literature about the structure of the observed cluster, in particular whether the excess proton is on the surface^2^ or in the interior of the cluster,^5^ subsequent studies using vibrational spectroscopy, electronic structure calculations, and force field calculations^3–15^ have unambiguously established that the cluster observed experimentally in supersonic jet expansions is based on an (H_2_O)_20_ pentagonal dodecahedron with the excess proton localized on the surface (as a hydrated hydronium ion) and the “extra” water molecule located in the interior of the cluster, as proposed by Searcy and Fenn. The high stability of this structural form of H^+^(H_2_O)_21_ is derived from the fact that the water molecule occupying the interior of the cage is engaged in four hydrogen bonds. Although there is a very large number of isomers with this structural motif (there are over 30,000 isomers for the pentagonal dodecahedral (H_2_O)_20_ cluster alone^16^), the measured vibrational spectra in the OH stretch region of the cold H^+^(H_2_O)_21_ cluster and its H^+^(D_2_O)_21_ isotopomers appear to derive from a single isomer or from a small group of isomers with very similar spectra.^17^

Various H^+^(H_2_O)_21_ isomers with the dodecahedral structural motif can be divided into classes distinguished by the locations of the free OH groups and by the H-bonding arrangements of the central water monomer and the water monomers directly bonded to the hydronium ion. There has been considerable theoretical work classifying different classes of isomers of dodecahedral (H_2_O)_20_ (see, for example, ref 17). The lowest-energy class of isomers of H^+^(H_2_O)_21_, denoted here as Class I, is comprised of 10 isomers, the structures of which have been reported previously.^13^ These ten isomers are predicted to be very close in energy, lying within 0.3 kcal/mol of one another.^13^ Figure 1 displays the structures of two of the Class I isomers. The structure on the left (labeled I-4) is the lowest-energy isomer found in the B3LYP^18–20^ calculations of Shin et al.,^1^ and the structure on the right (labeled I-1) is the lowest-energy isomer from the second-order Møller–Plesset perturbation theory (MP2)^21^ calculations [with corrections for vibrational zero-point energy (ZPE)] of Xantheas.^4^ To more clearly depict the H-bonding arrangements of these two isomers (as well as of the other isomers considered in this study), it is useful to consider two-dimensional representations such as those shown in Figure 2. From this figure, it is seen that these two isomers are similar structurally, with both having three pairs of adjacent (i.e., nearest neighbor) free OH groups (AFOH), three single-acceptor double-donor (ADD) water molecules bonded to the hydronium ion, and the interior water molecule participating in six 5-membered rings. It is also seen that the directions of the H-bonds of six water molecules in a six-membered ring involving the interior water molecule must be changed to interconvert I-4 and I-1. In a separate study, we will consider the pathways for isomerization of Class I isomers, but for the purpose of the current study, we simply note that these barriers are quite high (being over 9 kcal/mol).

**Figure 1:**
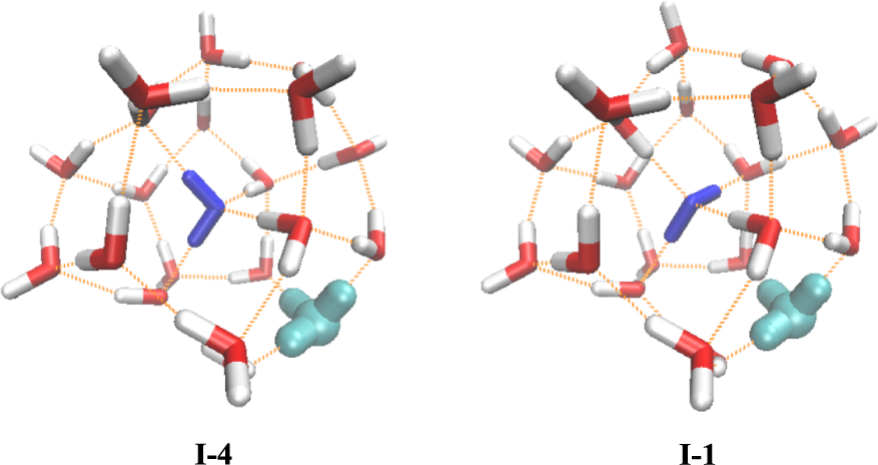
Lowest-energy isomers of H^+^(H_2_O)_21_ reported in the studies of Shin et al.^1^ (I-4) and Xantheas (I-1).^4^ The hydronium ion is colored in cyan, while the central water molecule is represented in blue.

**Figure 2:**
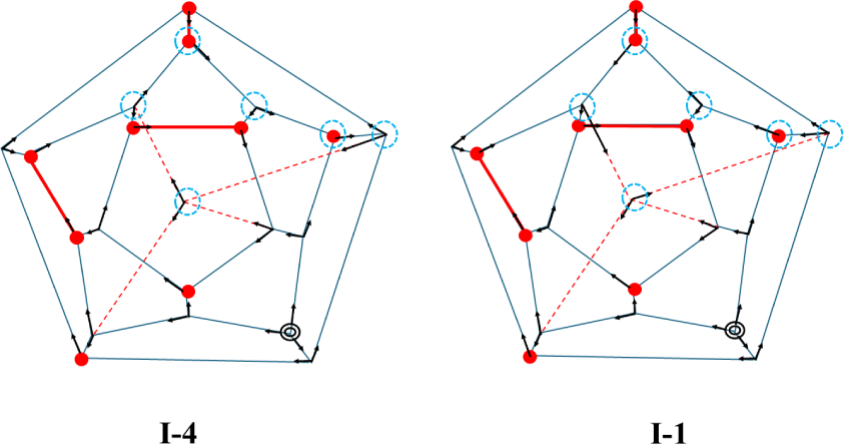
Two-dimensional representations of the I-4 and I-1 isomers of H^+^(H_2_O)_21_. The red dots indicate water molecules with dangling OH groups, and the double circle indicates a hydronium ion. The black arrows indicate the hydrogen bond directions, and the red solid lines denote adjacent free OH groups (AFOH). The six water molecules that have different hydrogen bond orientations in the two isomers are indicated with dashed circles.

To understand the evolution of the vibrational spectra of H^+^(H_2_O)_21_ and its H^+^(D_2_O)_21_ isotopomers with increasing temperature, it is necessary to have information on the energies of other low-energy classes of isomers. In this work, we elucidate the structures and energies of ten additional low-energy classes of isomers of H^+^(H_2_O)_21_ derived from the (H_2_O)_20_ pentagonal dodecahedron with an interior water molecule and the excess proton on the surface. We distinguish these classes by (1) the number of adjacent free OH groups (AFOH), (2) the number of dangling OH groups associated with water molecules directly attached to the hydronium ion (DHNH), (3) the number of AADD water molecules bonded to the hydronium ion (AADDH), (4) the H-bonding environment of the interior water monomer, and (5) the number of H-bonds in which the hydronium ion is engaged. We also consider two classes of isomers, denoted here as Classes XII and XIII, in which four 5-membered rings on the surface of the cluster are replaced by fused 4- and 6-membered rings, as shown in Figure 3. These isomers are more stable than those containing only a single 4-membered and a single 6-membered ring on the surface of the cage. As shown in Figure 4a, the latter includes surface water molecules involved in two and four hydrogen bonds with other surface molecules. This arrangement is energetically unfavorable compared to that with two 4-membered and two 6-membered rings, where all water molecules in these rings are engaged in three hydrogen bonds with other surface molecules. Clusters with two 4-membered and two 6-membered rings (arranged as shown in Figure 4b) have been identified in calculations by Hodges and Wales^8^ using the force field of Kozack and Jordan.^22^

**Figure 3:**
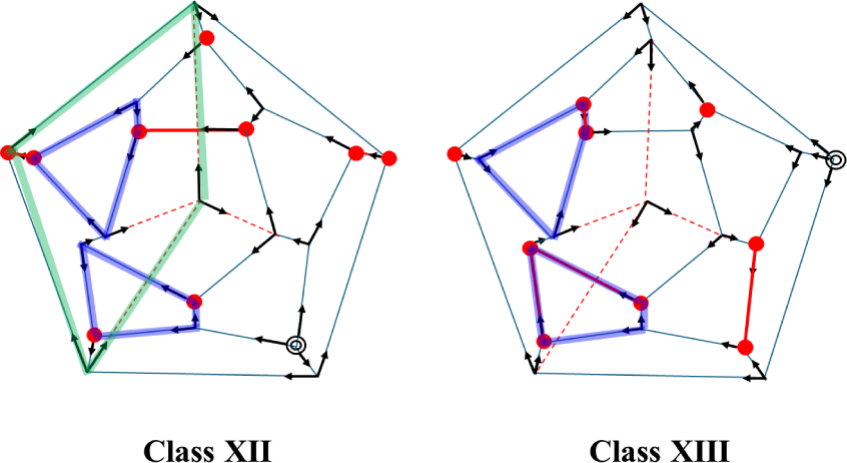
Two-dimensional representations of the lowest-energy isomers in Classes XII and XIII. The symbols indicating the different H-bonding environments are defined in Figure 2. The 4-membered rings involving the surface molecules are marked with blue lines, and the 4-membered ring involving the internal water molecule in the Class XII isomer is highlighted with green lines.

**Figure 4:**
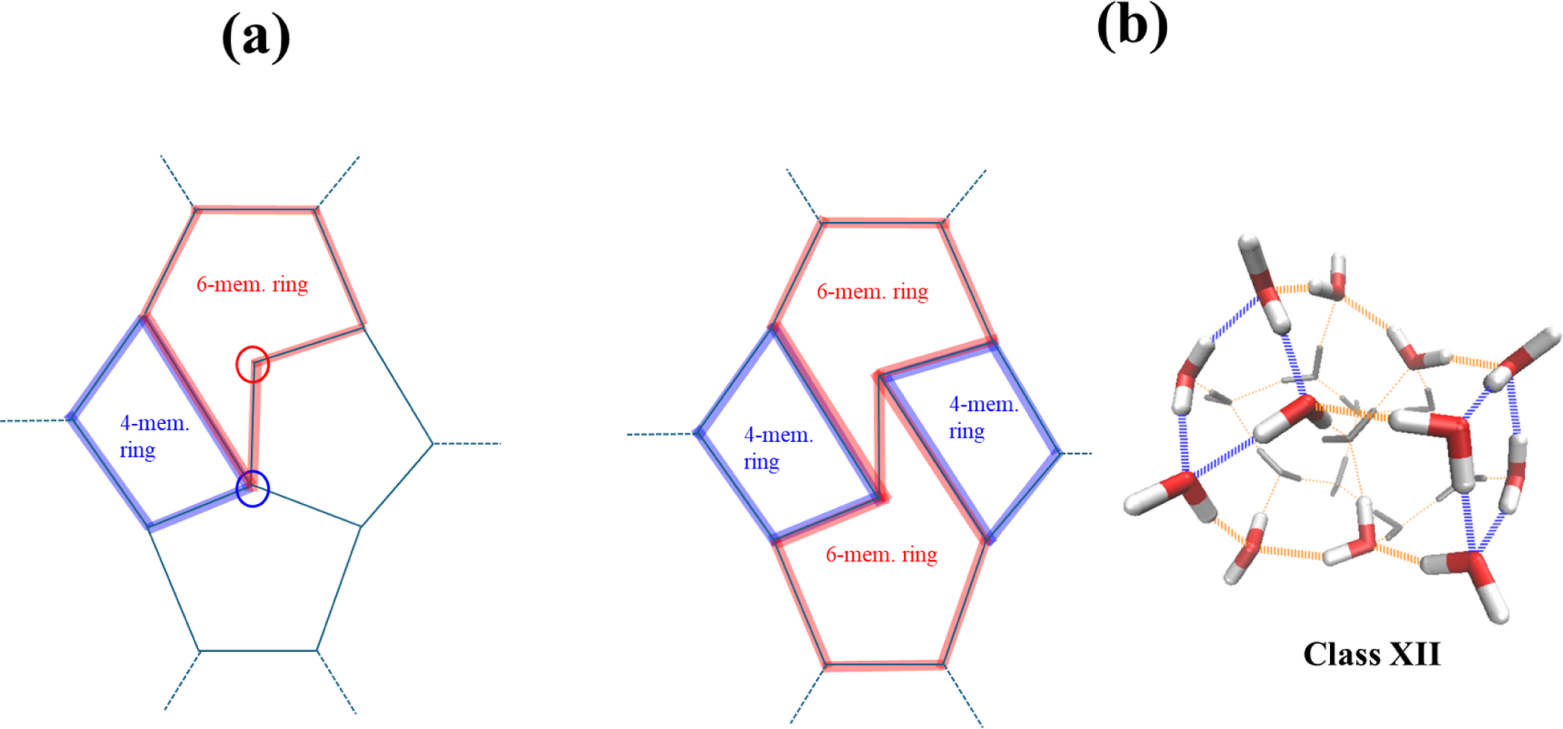
(a) Connectivity of clusters with one 4-membered and one 6-membered ring. The red and blue circles indicate, respectively, water molecules engaged in two and four H-bonds to other molecules on the surface. (b) Connectivity of clusters with two 4-membered and two 6-membered rings along with the 3D structure of the lowest-energy isomer of Class XII exhibiting this feature.

All possible isomers in each class were enumerated, and their geometries were optimized using the B3LYP density functional with D3 dispersion corrections.^23^ The structures of the ten lowest-energy isomers of each class, as determined from the B3LYP+D3 calculations, were reoptimized using the resolution-of-the-identity second-order Møller–Plesset perturbation theory (RI-MP2).^24^ The resulting geometries were employed for single-point calculations with larger basis sets, including higher-order correlation effects through the DLPNO-CCSD­(T) method.^25^ For the lowest-energy isomer in each class, harmonic vibrational frequencies were calculated using the RI-MP2 method. Additional details of the calculations are provided in the next section.

## 2 Isomer Classification and Computational Details

### 2.1 Computational Details

We wrote a computer program to generate the initial structures of all isomers of H^+^(H_2_O)_21_ for each of the 13 classes considered. Each of these structures was optimized at the Becke3LYP+D3 level using the 6–311+G­(2d,p) basis set.^26–28^ For the 10 lowest-energy isomers in each class, as determined from the B3LYP+D3 calculations, geometries were reoptimized using the RI-MP2 method with the aug-cc-pVDZ basis set.^29,30^ These geometries were then employed in single-point RI-MP2 calculations with the aug-cc-pVTZ and aug-cc-pVQZ basis sets^20,21^ as well as in DLPNO-CCSD­(T) calculations with both the aug-cc-pVDZ and aug-cc-pVTZ basis sets. The B3LYP+D3 calculations were performed using Gaussian 16 ^31^ and the RI-MP2 and DLPNO-CCSD­(T) calculations were carried out using ORCA.^32^

### 2.2 Isomer Classes

Figure 5 shows the H-bonding arrangements of the lowest-energy H^+^(H_2_O)_21_ isomers in Classes I–XIII as determined at the RI-MP2/aug-cc-pVDZ level of theory. As noted above, the isomers in Class I have three AFOH, zero DHNH, and zero AADDH bonds, and a 5^6^ bonding arrangement of the internal water molecule. The higher-energy Classes II–XIII differ from Class I in one or more of these criteria. The distinguishing features of the various classes are summarized in Table 1. Significantly, it is energetically unfavorable to have adjacent dangling OH groups, dangling OH groups on the water molecules bonded to the hydronium ion, or bonding arrangements other than 5^6^ for the interior molecule. An example of the latter is Class IV, in which the internal water molecule is in a 45^4^6 ring arrangement, where it is engaged in one 4-membered ring, one 6-membered ring, and four 5-membered rings. Table 1 also indicates the nature of the rings of the surface water molecules. All isomers in Classes I–XI have 12 5-membered rings on the surface, while the isomers in Classes XII and XIII have two 4-membered rings, two 6-membered rings, and eight 5-membered rings on the surface. For Classes X and XI, the hydronium exhibits an ADD arrangement, whereas for the other classes it exhibits a DDD arrangement.

**Figure 5:**
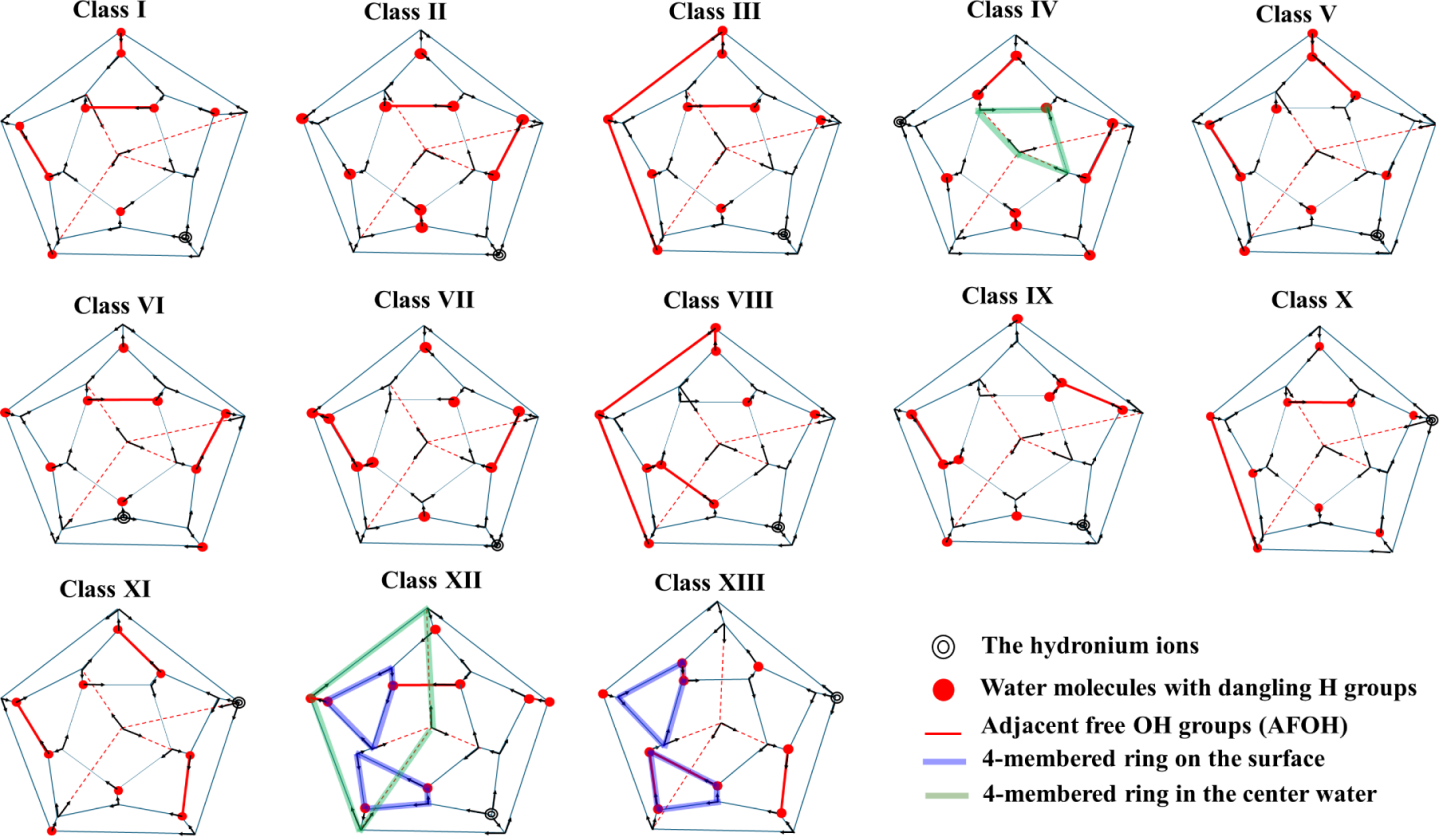
Two-dimensional representations of the lowest-energy isomers in Classes I–XIII. The symbols indicating the different H-bonding environments are defined in Figure 2. For the isomers from Classes IV and XII, the 4-membered ring involving the internal water molecule is highlighted with green lines. In Classes XII and XIII, two 4-membered rings on the surface are marked with blue lines.

**Table 1:** Characterization of the 13 Low-Energy Classes of Isomers of H^+^(H_2_O)_21_ Considered Here, and the Relative Energies of the Most Stable Isomers in Each Class

	Distinguishing features							Energy (kcal/mol)^a^	
Class	Surface water	Center water	AFOH	DHNH	AADDH	H_3_O^+^	# of isomers	B3LYP+D3	RI-MP2
I	5^12^	5^6^	3	0	0	DDD	10	0.00	0.00
II	5^12^	5^6^	3	0	1	DDD	45	1.09	1.40
III	5^12^	5^6^	4	0	0	DDD	58	1.16	1.25
IV	5^12^	45^4^6	3	0	0	DDD	33	1.22	0.90
V	5^12^	5^6^	3	1	0	DDD	550	1.36	1.83
VI	5^12^	5^6^	2	1	1	DDD	237	2.35	3.10
VII	5^12^	5^6^	4	0	1	DDD	1600	2.02	2.42
VIII	5^12^	5^6^	5	0	0	DDD	241	2.63	2.81
IX	5^12^	5^6^	4	1	0	DDD	1609	2.93	3.68
X	5^12^	5^6^	2	1	1	ADDD	74	3.46	3.87
XI	5^12^	5^6^	3	0	1	ADDD	162	4.38	4.41
XII	4^2^5^8^6^2^	45^3^6^2^	3	0	0	DDD	116	2.00	2.06
XIII	4^2^5^8^6^2^	5^5^6	4	0	0	DDD	556	2.10	2.15

a The B3LYP+D3 and RI-MP2 calculations were performed using the 6–311+G­(2d,p) and aug-cc-pVDZ basis sets, respectively.

## 3 Results and Discussion

Table 1 also reports the stabilities of the lowest-energy isomer in Classes II–XIII relative to those of Class I at both the B3LYP+D3/6–311+G­(2d,p) and RI-MP2/aug-cc-pVDZ levels of theory. Figure 6 shows the spread in the energies (using B3LYP+D3/6–311+G­(2d,p)) for all isomers in each class, while Figure 7 reports the relative energies of the 10 most stable isomers in each class, as calculated at the RI-MP2/aug-cc-pVDZ level.

**Figure 6:**
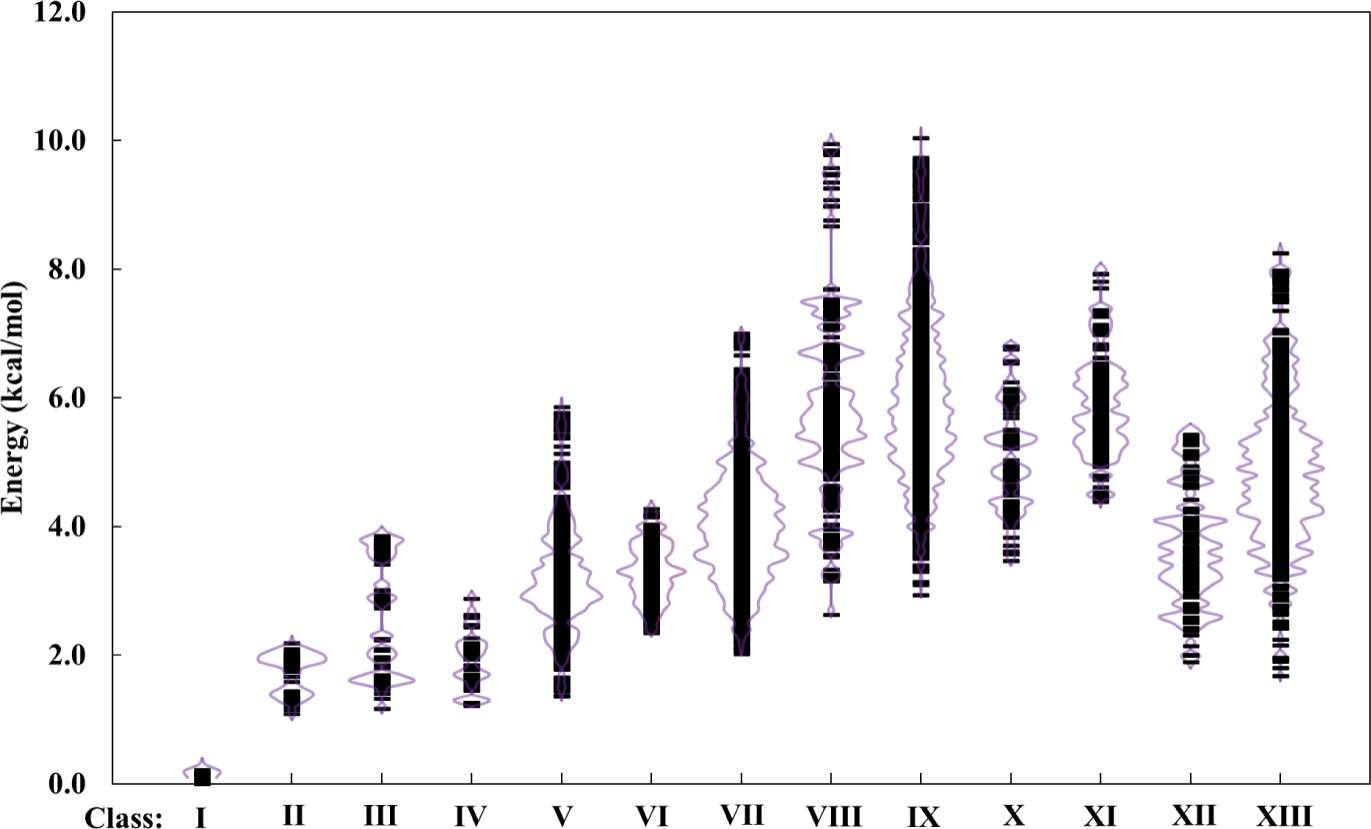
Energies of all isomers of H^+^(H_2_O)_21_ in each class relative to the most stable isomer at the B3LYP+D3/6–311+G­(2d,p) level of theory. The relative population densities as a function of energy for each class are represented by the solid purple lines.

**Figure 7:**
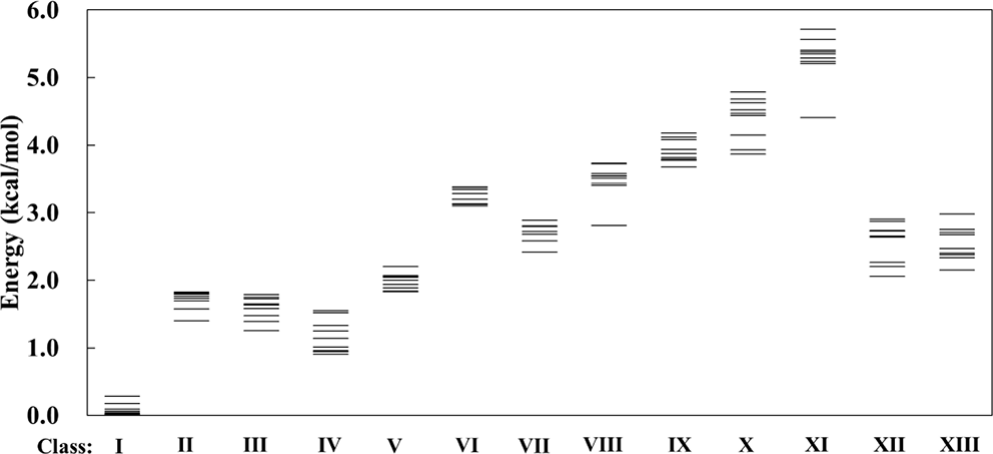
Energies of the 10 most stable isomers of H^+^(H_2_O)_21_ in each isomer class relative to the most stable isomer at the RI-MP2/aug-cc-pVDZ level of theory.

The 10 isomers of Class I are calculated at the RI-MP2/aug-cc-pVDZ level to lie energetically within 0.28 kcal/mol of one another. There is a total of 136 isomers in Classes II–IV. The lowest-energy isomers in these classes are predicted at the RI-MP2/aug-cc-pVDZ level to lie only 0.90 to 1.40 kcal/mol above the global minimum Class I isomer. There are 4473 isomers in Classes V–XI. These isomers are calculated at the RI-MP2/aug-cc-pVDZ level to start 1.83 to 4.41 kcal/mol (depending on the class) above the global minimum. We identified 116 and 556 isomers in Classes XII and XIII, respectively. At the RI-MP2/aug-cc-pVDZ level of theory, these two classes, which have two 4-membered and two 6-membered rings on the surface, start energetically at 2.06 and 2.15 kcal/mol above the global minimum isomer.

The H-bonding arrangements of the ten isomers in Class I, which were reported in ref 13, are depicted in Figure 8. These isomers can be divided into two subclasses with different orientations of the internal water molecule (assuming a fixed location of the hydronium ion). Isomers I-1, I-2, and I-3 belong to one subclass, and isomers I-4 through I-10 belong to the other subclass. As seen from Table S1, the most stable isomer in each subclass depends on the basis set employed and the method used to recover correlation effects (i.e., RI-MP2 vs DLPNO-CCSD­(T)). Although all 10 of these isomers are very close in energy in the absence of corrections for vibrational zero-point energy (ZPE), the inclusion of ZPE (calculated at the RI-MP2/aug-cc-pVDZ level) destabilizes the isomers in the second subclass relative to the lowest-energy isomer in the first subclass by 0.29–0.46 kcal/mol (see Table S1).

**Figure 8:**
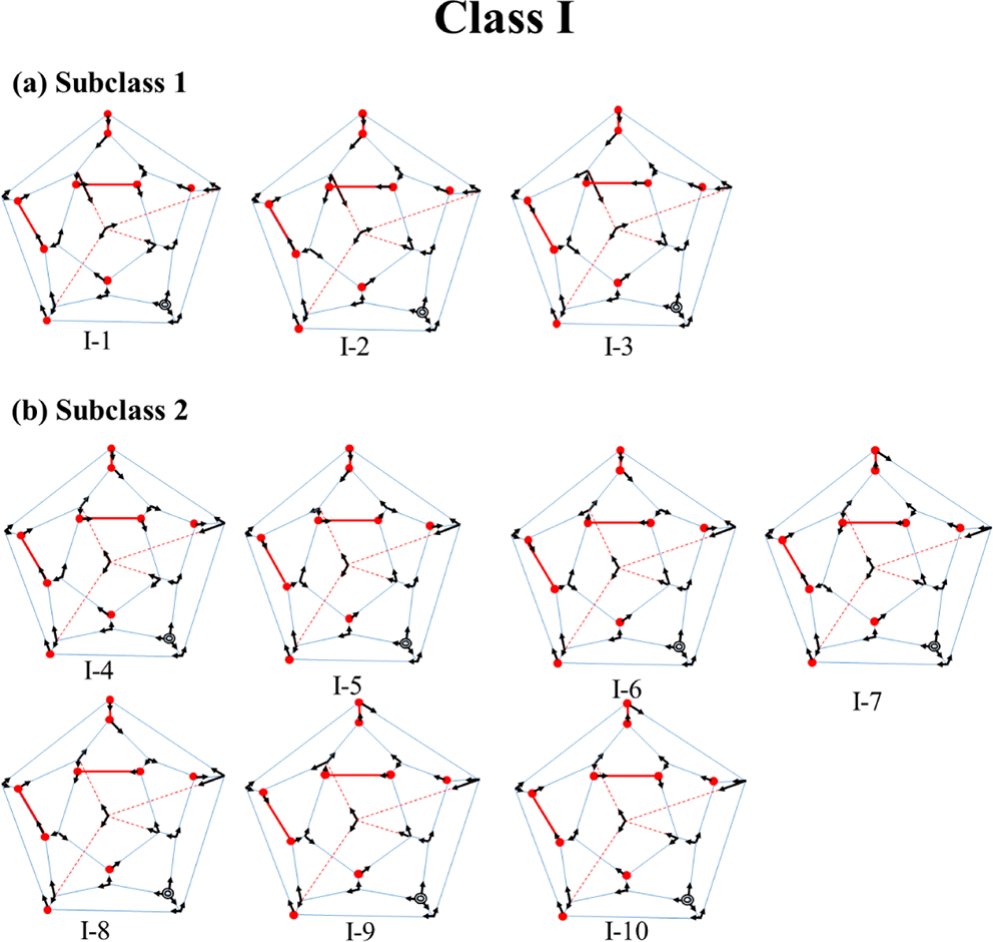
Two-dimensional representations of the structures of the 10 Class I isomers. (a) I-1 to I-3 belong to one subclass and (b) I-4 to I-10 belong to the other subclass.

With the exception of the isomer classes with a dangling OH adjacent to H_3_O^+^ (DHNH), the B3LYP+D3 and RI-MP2 calculations give relative energies that agree to within 0.4 kcal/mol. Although the B3LYP+D3 calculations predict that the most stable isomers from Classes II–IV are close in energy, the RI-MP2/aug-cc-pVDZ calculations predict that the lowest-energy isomer from Class IV is 0.3 kcal/mol lower in energy than the most stable isomers from Classes II and III, and only 0.90 kcal/mol higher than the most stable isomer from Class I. We note also that the MP2/aug-cc-pVDZ calculations predict the classes with a dangling OH adjacent to the H_3_O^+^ (V, VI, IX, and X) to be significantly (0.4–0.8 kcal/mol) less stable relative to the global minimum than predicted by the B3LYP+D3 calculations. The most stable isomers from Classes XII and XIII are predicted at the RI-MP2/aug-cc-pVDZ level to be about 2.1 kcal/mol less stable than the global minimum isomer.

We now consider the impacts of expanding the basis set from aug-cc-pVDZ to aug-cc-pVTZ and aug-cc-pVQZ, as well as including higher-order correlation effects by means of the DLPNO-CCSD­(T) method. The results of these calculations are summarized in Table 2. At the RI-MP2 level of theory, the relative energies of the most stable isomers in Classes I–IX remain largely unchanged when adopting the more flexible aug-cc-pVTZ and aug-cc-pVQZ basis sets. For these isomers, the relative energies calculated using the RI-MP2 method are impacted by at most 0.12 kcal/mol when transitioning from the aug-cc-pVDZ to the aug-cc-pVTZ basis set, and by at most 0.07 kcal/mol when transitioning from the aug-cc-pVTZ to the aug-cc-pVQZ basis set. In contrast, the lowest-energy isomers in Classes XII and XIII are stabilized, and the lowest-energy isomers in Classes X and XI are destabilized (at the RI-MP2 level) by about 0.3 kcal/mol relative to the most stable Class I isomer when transitioning from the aug-cc-pVDZ to the aug-cc-pVTZ basis set. The Class XII and XIII isomers are further stabilized at the RI-MP2 level by 0.09–0.15 kcal/mol when transitioning to the aug-cc-pVQZ basis set. We also explored extrapolating the RI-MP2 energies to the complete basis set (CBS) limit.^33^ This was accomplished using the three-point extrapolation formula of ref 33, as applied to the energies obtained using the aug-cc-pVDZ, aug-cc-pVTZ, and aug-cc-pVQZ basis sets. The relative energies at the RI-MP2 level were altered by at most 0.09 kcal/mol by this extrapolation to the CBS limit.

**Table 2:** Relative Stability (in kcal/mol) of the Lowest-Energy Isomers of H^+^(H_2_O)_21_ in Classes I–XIII, with Increasing Basis Set Size and Inclusion of High-Order Correlation Effects

	RI-MP2				DLPNO-CCSD(T)			
Class	aVDZ^a^	aVTZ^a^	aVQZ^a^	CBSest^b^	aVDZ^a^	aVTZ^a^	CBS_est_ ^b^	CBS_est_ (zpe)
I	0.00	0.00	0.00	0.00	0.00	0.00	0.00	0.00
II	1.40	1.41	1.47	1.55	1.54	1.63	1.78	1.85
III	1.25	1.22	1.20	1.18	1.21	1.22	1.19	1.32
IV	0.90	0.94	0.92	0.89	0.95	0.88	0.83	0.59
V	1.83	1.74	1.75	1.79	2.06	1.87	1.91	1.87
VI	3.10	2.98	3.01	3.08	3.59	3.38	3.49	3.61
VII	2.42	2.51	2.58	2.64	2.50	2.56	2.69	2.90
VIII	2.81	2.82	2.81	2.81	2.70	2.74	2.73	2.63
IX	3.68	3.57	3.55	3.55	3.85	3.73	3.71	3.53
X	3.87	4.11	4.22	4.31	4.33	4.55	4.74	3.43
XI	4.41	4.79	4.86	4.88	4.35	4.77	4.87	3.98
XII	2.06	1.78	1.69	1.67	1.76	1.41	1.30	1.37
XIII	2.15	1.81	1.66	1.57	1.93	1.55	1.31	1.34

a aVDZ, aVTZ, and aVQZ refer to aug-cc-pVDZ, aug-cc-pVTZ, and aug-cc-pVQZ, respectively.

b The finite basis set results are extrapolated to the complete basis set (CBS) limit as described in the text.

As seen from the results summarized in Table 2, the relative energies of the most stable isomer (as determined at RI-MP2/aug-cc-pVDZ) in the various classes are impacted by up to 0.7 kcal/mol when transitioning from RI-MP2 to DLPNO-CCSD­(T). Most significantly, the most stable isomers in Classes IV, XII, and XIII are stabilized by 0.26–0.37 kcal/mol (relative to the most stable isomer from Class I) upon inclusion of high-order correlation effects. When correcting the DLPNO-CCSD­(T)/aug-cc-pVTZ energies by the changes in the MP2 energies from the aug-cc-pVTZ basis set to the extrapolated complete basis set limit, the most stable isomers in Classes XII and XIII are predicted to lie, respectively, only 1.30 and 1.31 kcal/mol above the most stable Class I isomer.

Although including harmonic vibrational ZPE corrections does not lead to large changes in the relative energies of the isomers from the various classes, they further stabilize the most stable isomer of Class IV relative to the most stable Class I isomer. Our best estimates, DLPNO-CCSD­(T) with corrections for extrapolation to the complete basis set limit and inclusion of vibrational ZPE, have the lowest-energy isomers from Classes IV, III, XIII, XII, II, and V lying, respectively, 0.59, 1.32, 1.34, 1.37, 1.85, and 1.87 kcal/mol above the global minimum isomer.

We now discuss the relevance of the results described above for the evolution of the vibrational spectra of H^+^(H_2_O)_21_ and H^+^(D_2_O)_21_ as a function of temperature. For temperatures up to about 150 K, the experimental vibrational spectrum of H^+^(H_2_O)_21_ displays a sharp, isolated band near 3700 cm^–1^ in the free OH region, a well-resolved band near 3570 cm^–1^ and broad, relatively featureless absorption from 3500 down to 3200 cm^–1^.^1,3,9^ The lack of well-resolved sharp structure in the 3200–3500 cm^–1^ region in the case of H^+^(H_2_O)_21_ is due to anharmonic effects coupling high- and low-frequency vibrations. These couplings are significantly suppressed in H^+^(D_2_O)_21_, for which the spectrum at temperatures of 120 K or lower displays three well-resolved bands between 3200 and 3500 cm^–1^ in addition to the two higher-energy lines mentioned above, reflecting OH groups in different H-bonding environments.^13^ The sharp structure below 3600 cm^–1^ persists up to *T* = 120 K but is largely washed out at *T* = 150 K. We note also that caloric measurements by Boulin and coworkers show that the heat capacity curve of H^+^(H_2_O)_21_ starts to rise rapidly near *T* = 140 K.^34^ Similar behavior was found in the heat capacity curve calculated by Korchagina et al.^35^ The latter authors employed parallel tempering molecular dynamics simulations using a self-consistent-charge density-functional tight-binding (SCC-DFTB) method^36,37^ to describe the energetics. Both the experimental vibrational spectra and the caloric curves suggest that a significant structural change occurs in the cluster at a temperature near 140 K. The 10 isomers in Class I have very similar calculated (RI-MP2/aug-cc-pVDZ) vibrational spectra (Figure S1). Thus, changes of the populations of the different isomers in Class I do not appear responsible for the washing out of the vibrational spectra as the temperature approaches 150 K. This suggests that there is a significant population of isomers other than those in Class I at temperatures of 150 K and above.

To explore how the vibrational spectra of the various classes differ, we calculated the B3LYP+D3/6–311+G­(2d,p) level vibrational spectra of all 40 D_3_O^+^(HDO) (D_2_O)_19_ isotopomers of the lowest-energy isomer from each of the 13 classes of isomers considered here. For each isomer, the spectra of the 40 isotopomers were added together (the isotopomers with the H on the hydronium ion were not considered, as their OH stretch frequencies are outside the spectral range of interest). This procedure is clearly not appropriate at very low temperatures, e.g., 10 K, where differences in the vibrational ZPEs for the various isotopomers are important, but it is appropriate for temperatures above 120 K, where the differences in the ZPEs would not appreciably impact the relative populations. The resulting spectra are shown in Figure S2. These spectra were generated by using Lorentzian line widths of 5 cm^–1^. As shown in ref 13 the vibrational spectrum of the class I isotopomers of H^+^(D_2_O)_21_ calculated in this manner is in good agreement with the measured spectra at temperatures below 120 K.

Using the energies of the isomers reported above, we calculated the Boltzmann factors, which provide the relative populations of the various isomer classes at *T* = 150 K. The populations were obtained as follows: the energies of the lowest-energy isomer in each class were taken from the CBS-estimated DLPNO-CCSD­(T) calculations, with the inclusion of vibrational ZPE corrections calculated at the B3LYP+D3 level, and the relative energies of the various isomers in each class were determined by the B3LYP+D3 calculations. These populations are shown in Figure 9, from which it is seen that there is a significant population of the lowest-energy isomer of Class IV at this temperature. The population of Class IV isomers at 150 K would likely be even higher if we allowed for all of the isomers in this class. From Figure 10, it is seen that the vibrational spectra of the lowest-energy isomers from Classes I and IV differ appreciably, consistent with the interpretation that the growing population of Class IV isomers with increasing temperature contributes to the smearing out of the experimental vibrational spectrum of H^+^(D_2_O)_21_ as the temperature approaches 150 K. This conclusion is consistent with the DF-TB MD simulations of ref 34, which indicated that there is a significant population of isomers with 4-membered rings in the melted cluster.

**Figure 9:**
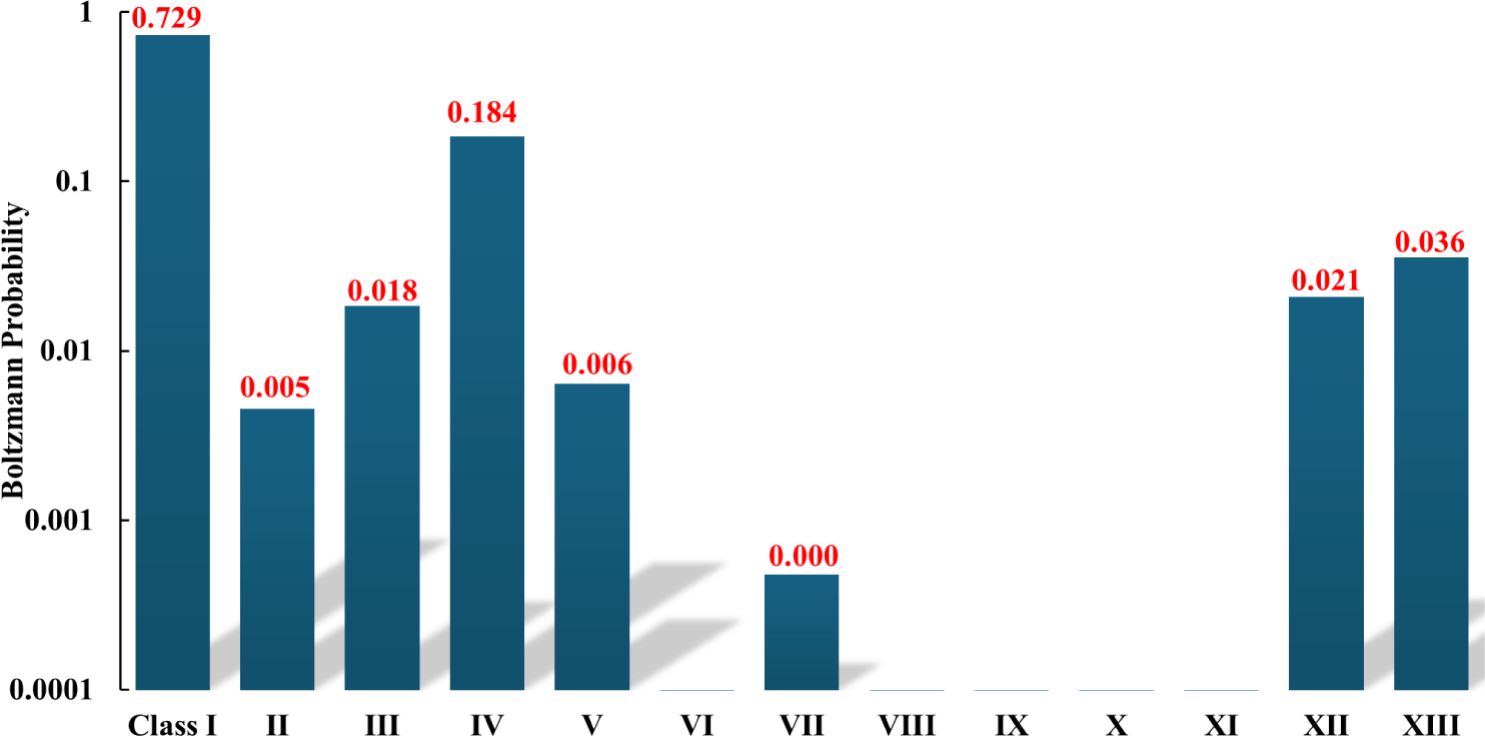
Approximate calculated Boltzmann probabilities for various H^+^(H_2_O)_21_ isomer classes at a temperature of 150 K.

**Figure 10:**
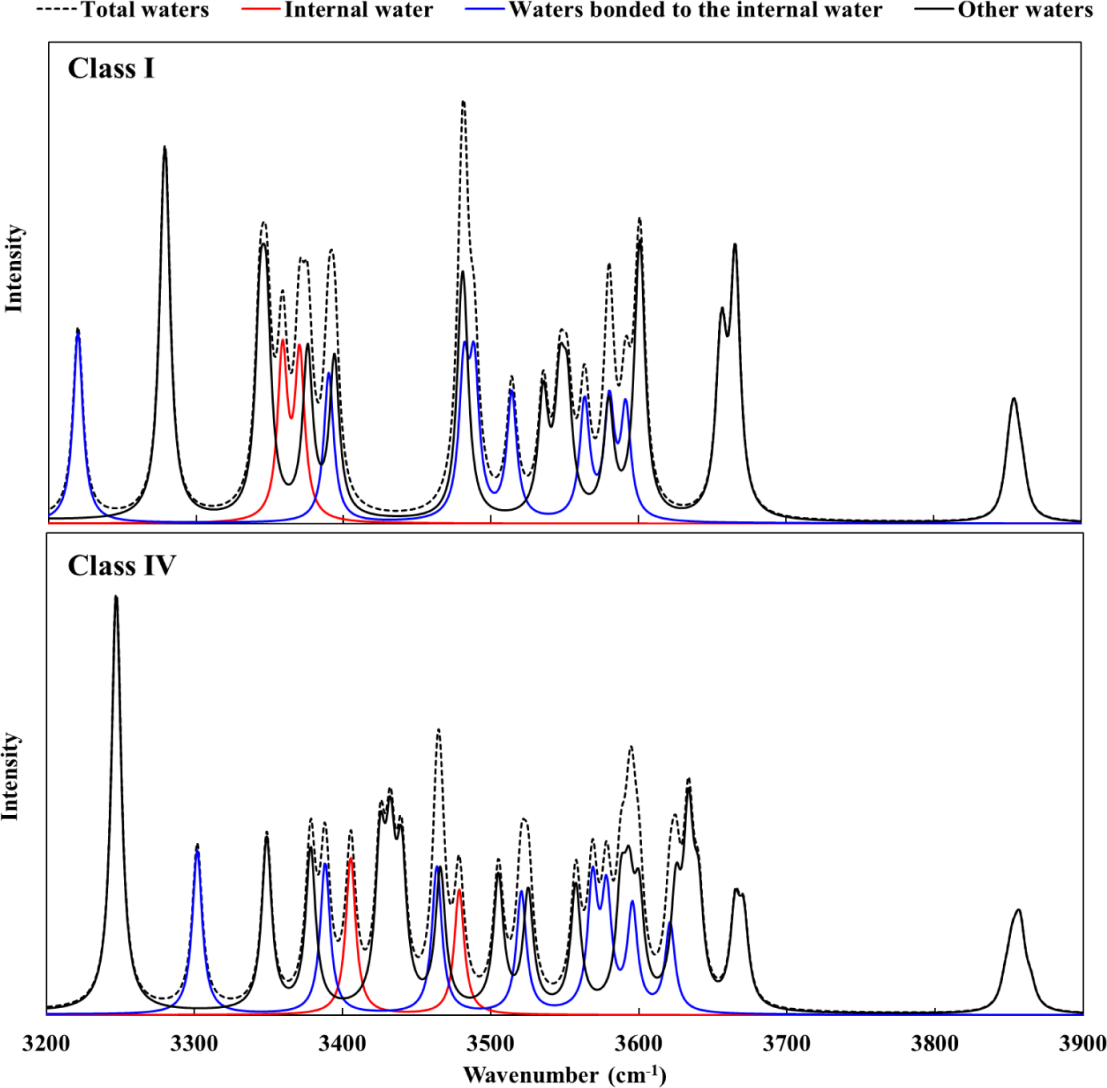
Sum of the calculated vibrational spectra of the 40 D_3_O^+^(HDO) (D_2_O)_19_ isotopomers of the lowest-energy isomers of Class I and Class IV using Lorentzian line widths of 5 cm^–1^. The vibrational spectra were calculated in a harmonic approximation at the B3LYP+D3/6–311+G­(2d,p) level. The contributions from HOD molecules in different environments are indicated by color coding.

## 4 Conclusions

In this work, we characterized the isomers in 13 low-energy classes of H^+^(H_2_O)_21_ isomers derived from the pentagonal dodecahedral (H_2_O)_20_ cage with an interior water and the excess proton on the surface of the cluster. Eleven of these classes of isomers retain the pentagonal dodecahedral arrangement of surface molecules, but in two of the classes, four of the surface pentamers are converted to two 4-membered and two 6-membered rings. There are 10 isomers in the lowest-energy class, which are predicted to lie within 0.3 kcal/mol of one another in the absence of corrections for vibrational ZPE. For the Class I isomers and for 10 of the other classes, the interior water participates in six 5-membered rings. However, in the second most stable class of isomers, denoted here as Class IV, the interior water is engaged in a 4-membered ring. As a result, the vibrational spectra of the Class IV isomers differ appreciably from the spectra of the Class I isomers. Our calculations predict that there should be a sizable population of Class IV isomers at *T* = 150 K, which is consistent with the loss of sharp structure in the vibrational spectra of H^+^(D_2_O)_21_ as the temperature approaches 150 K.

## Supplementary Material



## Notes

Calculated vibrational spectra of the 10 Class I isomers of H^+^(H_2_O)_21_; the sum of the spectra of the 40 D_3_O^+^(HDO) (D_2_O)_19_ isotopomers corresponding to the lowest-energy isomer from each of the Classes I–XIII; relative energies of the 10 Class I isomers of H^+^(H_2_O)_21_ at various levels of theory; the coordinates of these isomers and lowest-energy isomers of each of the other classes considered (PDF)
