# Supplementary material for: Low-Energy Isomers of the Magic Number H+(H2O)21 Cluster
Source: J Phys Chem A. 2025 May 21;129(22):4927–35. doi: 10.1021/acs.jpca.5c01977 (PMC12147204; doi:10.1021/acs.jpca.5c01977)
Supplement: Supplementary file 1 [file jp5c01977_si_001.pdf]

Supporting Information for

Low-Energy Isomers of the Magic Number $H^+(H_2O)_{21}$ Cluster

*T.-H. Choi, E.V. Henderson, and K. D. Jordan**

Department of Chemistry, University of Pittsburgh, Pittsburgh, PA 15260.

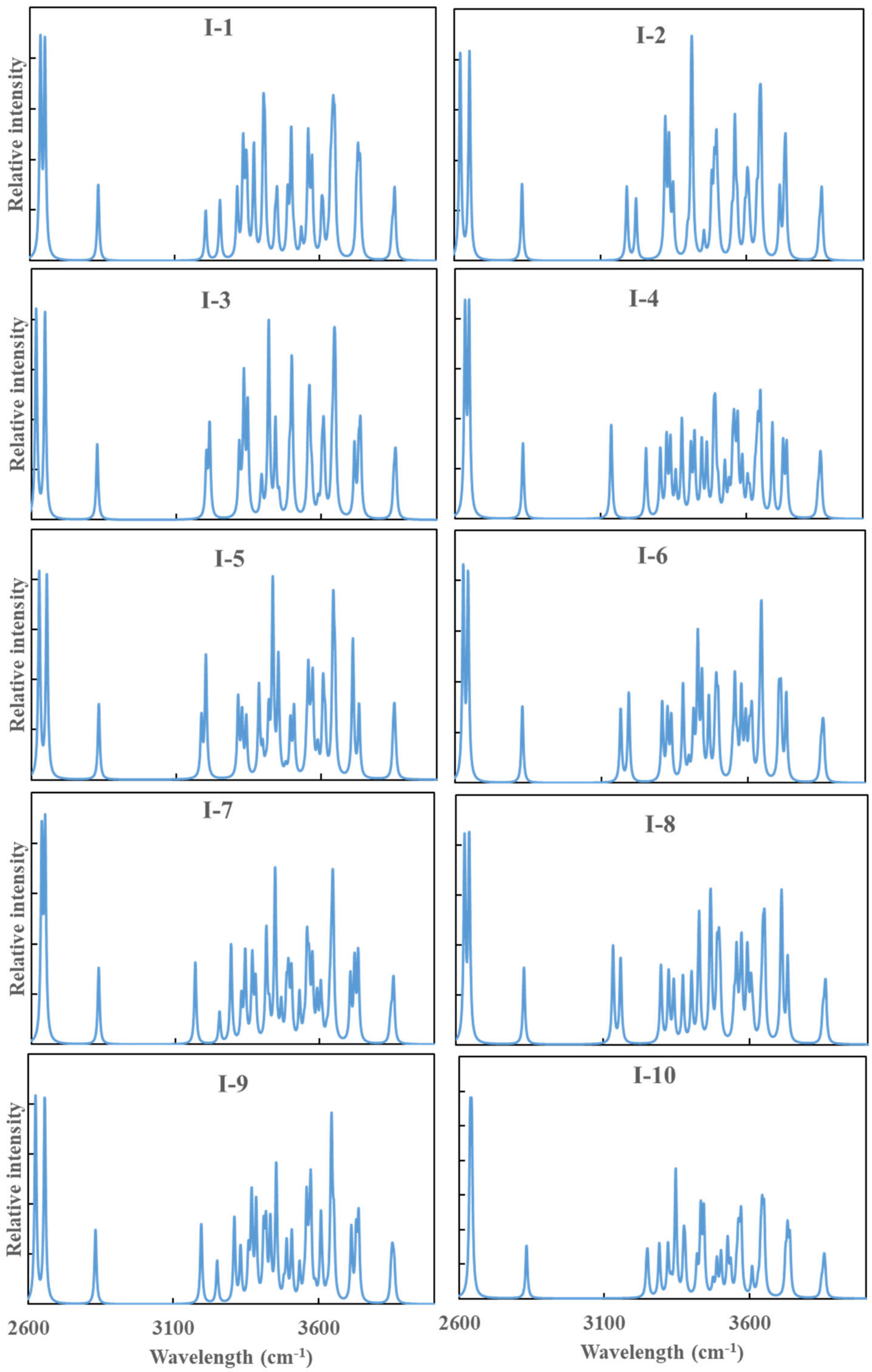

Figure S1. Harmonic vibrational spectra for the ten Class I isomers of $H^+(H_2O)_{21}$ computed using the RI-MP2 method in conjunction with the aug-cc-pVDZ basis set. 5 cm^{-1} Lorentzian line widths were assumed.

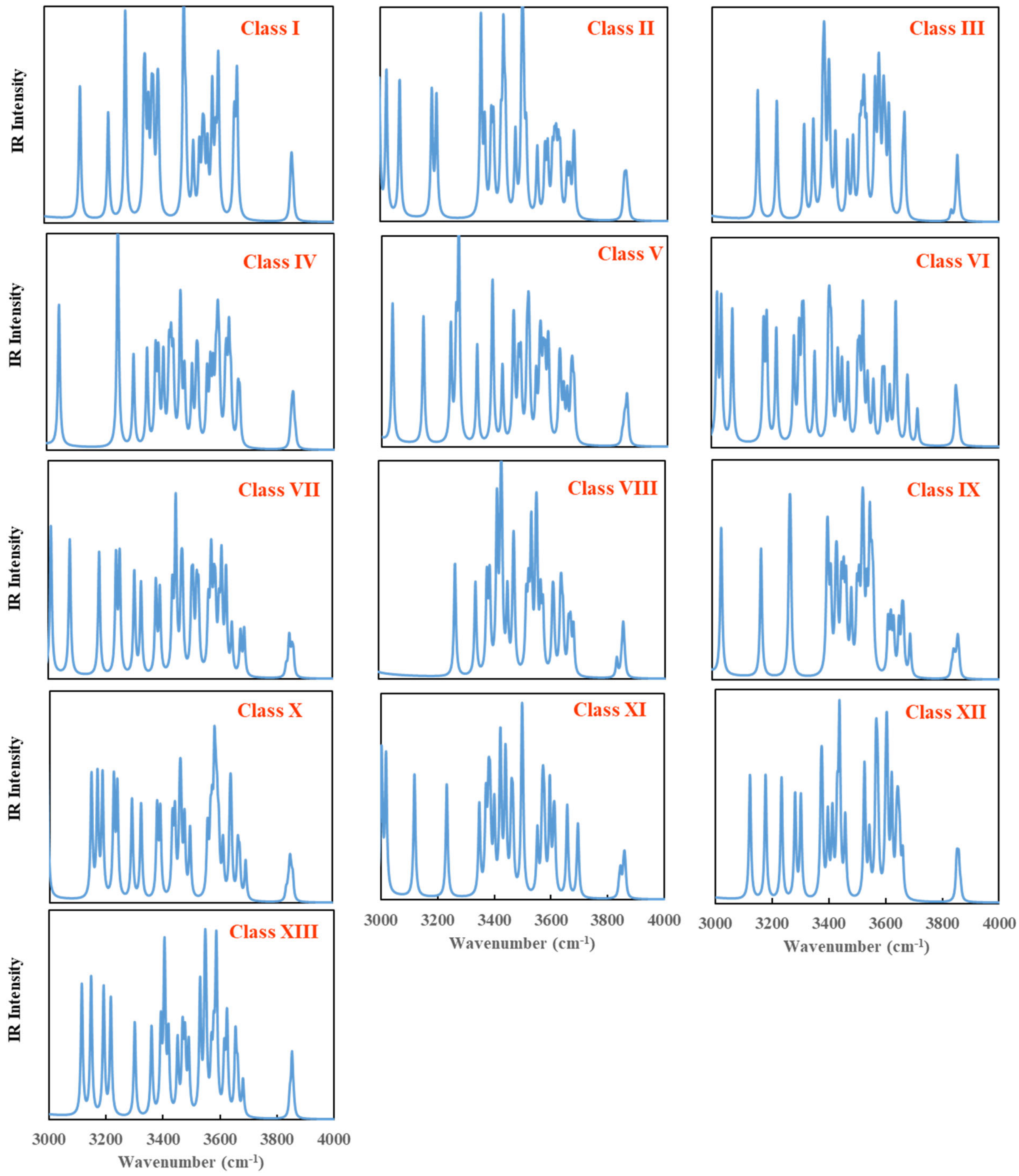

Figure S2. Sum of the spectra of the 40 $D_3O^+(HDO)(D_2O)_{19}$ isotopomers of the lowest energy isomer from each of the Classes I – XIII. The vibrational spectra were calculated at the B3LYP+D3/6-311+G(2d,p) level. 5 cm^{-1} Lorentzian line widths were assumed.

Table S1: Relative energies (kcal/mol) of the ten Class I isomers of $H^+(H_2O)_{21}$ at various levels of theory. The lowest energy isomer in each case is indicated in red.

		B3LYP+D3		RI-MP2					
		6-311+G(2d,p)		aug-cc-pVDZ		aug-cc-pVTZ		aug-cc-pVQZ	
	Isomers	E	E+ZPE	E	E+ZPE	E	E+ZPE	E	E+ZPE
Subclass 1	I-1	0.21	0.10	0.09	0.00	0.02	0.00	0.06	0.00
	I-2	0.13	0.00	0.01	0.10	0.03	0.18	0.02	0.14
	I-3	0.11	0.02	0.00	0.03	0.00	0.10	0.00	0.07
Subclass 2	I-4	0.00	0.03	0.04	0.29	0.00	0.32	0.01	0.29
	I-5	0.04	0.04	0.02	0.27	0.03	0.35	0.01	0.30
	I-6	0.08	0.06	0.06	0.31	0.08	0.40	0.06	0.33
	I-7	0.14	0.11	0.18	0.40	0.15	0.44	0.14	0.39
	I-8	0.00	0.00	0.03	0.30	0.07	0.41	0.04	0.34
	I-9	0.17	0.10	0.17	0.36	0.11	0.37	0.10	0.32
	I-10	0.20	0.18	0.28	0.53	0.18	0.49	0.18	0.46

Geometries (*xyz* format) obtained from RI-MP2/aug-cc-pVDZ calculations

1. Ten Class I isomers of $H^{+}(H_2O)_{21}$.

I-1

O	2.52880453553439	-1.91414247712372	-2.13694362791673
H	2.64712003865472	-1.72659493291225	-1.18144532319947
H	2.63175978266906	-1.04132583652139	-2.56693631678219
O	-0.07142718659879	0.80934343350993	-2.57845210354587
H	-0.46566676436844	1.70406419388876	-2.62092135410199
H	0.84561462700325	0.89160496430449	-2.92137581625644
O	3.59695092463886	0.89099961065937	1.61352834551731
H	4.54476074680620	0.79030341277813	1.77706633112350
H	3.28522121541686	0.01804288230853	1.25431230406124
O	-2.10974367179167	-1.71403100770734	0.50214041100619
H	-2.48581324275602	-1.47626857239705	1.37360688579493
H	-2.82330882623630	-1.56659120926944	-0.15619579835064
O	2.37492333953318	-1.23607631405265	0.56231041298343
H	1.48536671178479	-0.83264265711528	0.40854962343726
H	2.19140807942136	-2.01108463442756	1.13593225866657
O	-2.88063655574340	1.92838241676956	2.01293313775077
H	-1.91547690744179	2.14353469764238	1.99877818164927
H	-3.31900523142534	2.68099137990280	2.43276031088912
O	2.62456105535420	0.77169825513201	-3.02974664195743
H	2.84071116391257	1.32962714981642	-2.23796011547449
H	3.20075647474968	1.07945385810196	-3.74175800745867
O	-3.66085415824464	-0.95704668290856	-1.62280059546331
H	-4.57307439773217	-1.10615006850260	-1.90415997884245
H	-3.58513660869291	0.01175466901892	-1.42670240672756
O	-1.39089021637093	-1.53608361009796	-3.20196056720771
H	-0.98607503425018	-0.64537271709746	-3.13542856471369
H	-2.25263771121189	-1.44334858760568	-2.74811636072551
O	0.60528858309022	3.93456869101714	-0.20620250944382
H	0.78325866692142	4.82524702008095	0.12529380929450
H	0.31214945738998	3.40385433285910	0.57581999682731
O	-1.28848940453124	3.26176056607158	-2.10767512136821
H	-0.64509860608113	3.67556117300476	-1.48977945029822
H	-1.56696728911300	3.96274954356080	-2.71214367660286
O	2.89960886146964	2.25645926450186	-0.82898934133861
H	3.18317906815598	1.90497338359441	0.03526669672797
H	2.18279892017007	2.88909218507813	-0.64221603616957
O	-3.23986361808955	1.56345725502181	-0.84737510540093
H	-2.64355090138362	2.21709416123450	-1.25576643031361
H	-3.20084925675563	1.72708158860317	0.11226242515190
O	1.65121341595516	1.24162407573616	3.51171737937758
H	2.46908388031245	1.15666359588211	2.96715928131141
H	1.93440261537032	1.61152880428592	4.35847885468981
O	1.32701179438677	-3.23682186089461	2.15806707738116
H	1.68475079206147	-3.88209769885369	2.78173829634356
H	0.82357425793083	-2.57679829454786	2.69711054093451
O	-0.20525876046138	2.12717210319031	1.64467267217439
H	-0.13068263607579	1.32306680138266	1.06186491110011
H	0.41542822646885	1.94459135079740	2.38269864068149

O	0.08112147969129	-1.18346028118178	3.32639405764507
H	0.58524839840140	-0.36883940363698	3.50094685857531
H	-0.86197759680155	-0.93875569190390	3.32857720303849
O	-0.39014355023372	-3.82549104472573	-0.00291482274450
H	0.24994592143838	-3.78335155306222	0.73561219880330
H	-1.09659236580746	-3.19632387009834	0.25495529577616
O	-2.71597886480360	-0.64844693804079	3.00321663383326
H	-3.35995541101219	-0.91671673373903	3.67218839065561
H	-2.92228584951758	0.28778635471242	2.78604093049762
O	-0.01311206407421	-0.01170863910271	0.03315034030459
H	-0.04815883601902	0.27218059854801	-0.91286992452036
H	-0.78603820395285	-0.61309408787529	0.16515531930724
O	0.34079493713330	-3.21361994527512	-2.36820426092855
H	1.22252243313828	-2.69057358288970	-2.32302409109168
H	-0.38783928451908	-2.57488570628517	-2.70783672016336
H	0.08730093713209	-3.49338394314459	-1.41245248420353

I-2

O	-1.98113951263267	-0.87196593176144	-3.15305958096027
H	-1.15175394089720	-1.32271077058691	-2.88569990702257
H	-1.68232985371794	0.01529312884631	-3.43662456096775
O	-1.26963995125140	2.19237176565747	-1.00537717047317
H	-0.82349656847363	2.99603347222857	-0.66939845253090
H	-1.17000990810556	2.20036392739332	-1.98264205287210
O	2.95040321337344	-1.23756307350775	-2.57018086314437
H	3.53036511238681	-1.61072451308583	-3.24744759411199
H	3.36204332799983	-1.46557823108252	-1.70603262669147
O	-1.44764276633763	-0.99168924197397	2.11070726140558
H	-0.81934879980525	-1.25338114206327	2.81414435931741
H	-2.01544131960179	-0.28627497059888	2.49157625082404
O	0.29422500187533	-1.89755808055018	-1.93854848183928
H	0.22782965140553	-1.21042019390095	-1.23138119662053
H	1.19122456300568	-1.77439205636042	-2.30921213890275
O	1.75037026888725	0.96378233977043	3.41319634166233
H	2.18697018545418	0.89928514410268	2.52773046729760
H	2.39397440890483	1.38254671130633	4.00092883898295
O	-0.77983630905320	1.64983373222555	-3.63890898615921
H	0.19293805850349	1.51849489362958	-3.49803520382527
H	-0.86747796544068	2.14020199856223	-4.46692441805343
O	-2.86063898283820	1.28141598228036	2.66671482849515
H	-3.42790592109607	1.58587712987280	3.38720388756126
H	-2.05915667515664	1.86569920648926	2.67726912510645
O	-3.64847288307273	1.16582601646996	-0.04483149245081
H	-2.89144353674717	1.68111170933195	-0.39579735855081
H	-3.53464184651952	1.20437036260947	0.92605959452352
O	2.65508364882310	2.94561309773109	-0.66920023428443
H	3.51952463724200	3.37711547939332	-0.70541023896532
H	2.76466909545586	2.15682015800295	-0.08237246054200
O	0.31593613664777	4.06895476519854	0.28951674136393
H	1.21134811975517	3.84212048197768	-0.04782559078923
H	0.27354083979382	5.03456973501262	0.30470080313180
O	1.78331307878552	1.35824353702523	-2.94402135234487
H	2.27527082872285	0.52334605243604	-2.84000502577503
H	2.12203359095351	1.95648911369963	-2.25399278649268
O	-0.53070179402907	2.57987294508484	2.60612108856000
H	-0.19546958397965	3.14129739464467	1.88348537856532

H	0.23759213686895	2.08343418457469	2.94180776215134
O	3.67473692900401	-1.70091626780246	0.04823807445176
H	2.96566464553107	-2.24013184104286	0.50059329646994
H	4.51384539805060	-2.08201339435049	0.34009862292735
O	-0.30003462377101	-4.01307407630331	-0.36928466148927
H	-0.24991034201245	-4.90646638012270	-0.73508639865566
H	-0.04008095574513	-3.39559790547348	-1.09523402978747
O	2.48713054725079	0.69073257977947	0.82139465239513
H	1.58021374049664	0.42019752069146	0.51402656951606
H	3.05509344233812	-0.07683568606509	0.59171327935306
O	1.72612245538968	-2.94716314877447	1.30621561634661
H	0.99455177256802	-3.41267980718952	0.85633691644778
H	1.37340386082789	-2.58354149024167	2.13862349310082
O	-2.70778257406183	-2.75981840018159	0.40056274717364
H	-1.97248422721693	-3.33087417951084	0.09924318779400
H	-2.31681226864734	-2.23666259279099	1.13235401866997
O	0.75685408342027	-1.61139850080074	3.66664308742275
H	0.83370444641327	-2.02948484907896	4.53480676496461
H	1.17773564158159	-0.72684857411168	3.75268630480595
O	0.01560155985686	0.00261978587935	0.01026556890691
H	-0.46088050269068	0.79151622648546	-0.34738429958866
H	-0.53110892860063	-0.32088117710795	0.76665871709124
O	-3.67770084637508	-1.08110987157983	-1.25427827193971
H	-3.02234722061570	-0.97278561042293	-2.03668981787950
H	-3.70228196141044	-0.19249186600724	-0.74059307894584
H	-3.29710685767052	-1.79577475396236	-0.61954031412994

I-3

O	0.10586883379748	-2.99203507447049	2.38012418187619
H	0.44210348287076	-2.10198888941450	2.61738097289203
H	0.76137999955683	-3.32564442333203	1.73492597423030
O	0.29526925195622	-2.34129002895347	-1.35863541920035
H	0.67905119756876	-2.14239671026212	-2.23661363620153
H	0.94289880063345	-2.91223314375599	-0.89015953014438
O	3.40237775612886	0.72184920512277	2.25729927117709
H	4.12790988054305	0.81913529366578	2.88829021913535
H	3.16467504077982	1.63267593902271	1.97292735985504
O	-2.61392097033289	0.78844503974578	-0.14248954551888
H	-2.64063671255608	1.75838319773779	-0.27429262400205
H	-3.04978597194147	0.38329361930809	-0.92366235439589
O	0.81465850717116	-0.31114976247877	2.60439116632440
H	0.55339152763203	-0.17366805844535	1.66118763166324
H	1.74713895770698	-0.01811109479329	2.64401202859416
O	-0.54610101893347	3.02332133156500	-2.55581027065608
H	-1.14737008559405	3.32028053805316	-1.83195073589961
H	-0.54618165270310	3.73976708119293	-3.20470202297716
O	2.04402418124074	-3.51191732107489	0.38379787599793
H	2.66020013964770	-2.73475396415491	0.38833959779871
H	2.60518682011822	-4.29835267928948	0.36829755328961
O	-3.30691997022462	-0.67217391518116	-2.34655253776869
H	-4.05472966713438	-0.69907467655783	-2.95768751903958
H	-2.52706162427814	-0.36432432458467	-2.87643277431117
O	-2.36849446655238	-2.88076964289475	-0.85735104709483
H	-1.43869053789494	-2.81856576031144	-1.16239315467282
H	-2.83637544727146	-2.19754931724623	-1.37828074046899
O	3.28472650141028	0.17569074630151	-2.30659892452571

H	4.08554739708138	0.56540507207870	-2.68271752719063
H	2.74603905372374	0.93089446131433	-1.96245692105384
O	1.31747147734099	-1.19976593928675	-3.67625746064559
H	2.12571395096244	-0.74530892890683	-3.34901048418360
H	1.55913988827489	-1.59361824143911	-4.52522848026100
O	3.49141459844726	-1.27367303342047	0.19974458728373
H	3.56504569072083	-0.57381827735568	0.87394543949986
H	3.51434665019689	-0.82004065434015	-0.66201338358950
O	-1.11258075453863	0.33302704693312	-3.48655705412882
H	-0.25936167970480	-0.11811543513925	-3.62101468123437
H	-0.90522722357829	1.25308082748506	-3.24329639163212
O	2.29682515079931	3.02759508679056	1.21409231647574
H	2.10353932432148	2.74808636872365	0.28473188687299
H	2.74909014410993	3.87962569767319	1.14669549717445
O	-1.17333527423231	1.15277419471249	3.69312847525605
H	-1.18256490613847	1.30715875364171	4.64727834405690
H	-0.37158909972394	0.60728952215845	3.50472217260970
O	1.54819539783753	1.88896213631309	-1.13288169823777
H	0.96668560449037	1.19181811213418	-0.72386381269791
H	0.92929640297166	2.39452689762748	-1.70330990585555
O	-0.54009080892831	3.16673542065591	1.79269534702312
H	-0.80236362092399	2.55327000879964	2.50713779171099
H	0.43273818330275	3.14572023862986	1.75440008637176
O	-3.06732584841495	-0.41830635869393	2.30104312485975
H	-2.50903688894920	0.09774930223298	2.91656664454100
H	-3.05031426652955	0.09831932130542	1.46732387224273
O	-2.06863740937532	3.49982656961241	-0.33224620145208
H	-2.66188739404911	4.24789474916083	-0.18159771533953
H	-1.48765045640331	3.44938706024070	0.47774772589691
O	0.01105485312651	0.00427529395785	0.01097745303354
H	0.09736576912496	-0.84667911284602	-0.48518201837987
H	-0.94009238865645	0.26242955559551	-0.06284507378724
O	-2.34560075301276	-2.78545812244202	1.69312553692298
H	-1.36101337459800	-2.90743447286394	1.95768188150268
H	-2.40152194217638	-2.82298647587024	0.66826681362947
H	-2.63714920024449	-1.84299584968688	1.98611081674877

I-4

O	3.04995928455245	2.24105247122289	-0.91668600223041
H	2.57350829686627	2.25039768349919	-0.05891774315102
H	2.40420651472043	2.61684738475159	-1.54861686773753
O	0.07162324192493	0.48838879817811	-2.68010022483594
H	0.01183769887659	0.30330258733220	-1.71087787302712
H	-0.81658099391285	0.25443374879112	-3.01753422239241
O	-0.56409031734355	3.47588882969420	1.92395753603005
H	-0.33567208504718	4.33645462272132	2.30128806413195
H	0.29475036645692	2.99215227404682	1.79551182870442
O	1.01051897645078	-2.50863551469336	0.53374864102236
H	0.44514911252557	-2.93067955371910	1.21163409030004
H	0.94130502609946	-3.05878906395393	-0.27730540709830
O	1.47866080942409	1.86671291299163	1.34536976343142
H	0.95991614294621	1.14789322169487	0.90654014992933
H	1.94429120460131	1.41300155624182	2.08161202941646
O	0.67831460428408	-3.48864262179191	-1.99220681201778
H	0.74067754995555	-4.36134986466674	-2.40247699947738
H	-0.27688279951707	-3.22406352588337	-2.04999988392676

O	-1.86056518396681	-2.67269074931216	-1.87444893157526
H	-2.20091693919367	-1.86416130576743	-2.29945376085848
H	-2.28628667675956	-2.71039703294932	-0.99867741930655
O	-0.00439608374454	-0.64370292983668	3.60147669125229
H	-0.70009175567707	0.03209238384736	3.51453143805350
H	-0.38635342037254	-1.48296103113272	3.28586267974695
O	2.21663133199728	-1.26874852392495	-2.84740748121075
H	1.45478828823482	-0.66530596446119	-2.97929849653731
H	1.79922683051810	-2.12331157308939	-2.61693631726711
O	-3.40557876960770	1.70419166923956	-1.24130679374523
H	-4.21469079114135	2.22802217226728	-1.31872887551705
H	-2.66642231977407	2.37151261690111	-1.13177374584296
O	-1.97675700611701	1.45926825913579	3.14510471090935
H	-1.52859569521548	2.28183062165366	2.83682423627651
H	-2.53537937168353	1.72854438213570	3.88685856365066
O	3.45870145724880	-1.32062758321992	1.02135394385815
H	3.29166153142769	-0.80234909900117	1.83390608621546
H	2.66046284280621	-1.88279623299915	0.92989379056796
O	-2.62337052504836	-0.18592212691860	-3.09571589325116
H	-3.03870447482957	0.50903831366488	-2.53624287915337
H	-3.13245511086206	-0.20442825958524	-3.91727921023128
O	0.01381381520129	-0.01650910747308	0.00958631101694
H	0.34426696168286	-0.92870908436238	0.20015760083836
H	-0.92006894365225	-0.01428919972959	0.31923886962311
O	-1.43180841795650	3.37858871058294	-0.81061347990079
H	-1.14339436155470	3.50956458577931	0.11227093116508
H	-0.62584032133742	3.40567394239709	-1.36303595611439
O	-0.89758305229479	-3.13183439770140	2.46691208671455
H	-0.98396414797674	-3.90034963026596	3.04701460752189
H	-1.73754993521519	-3.08012897038889	1.95829042776489
O	-3.01402276517153	-2.56949500103338	0.78443748870393
H	-3.91821503142127	-2.89592662883904	0.88874969859758
H	-3.06524064753314	-1.58632627315537	0.86637749345407
O	0.84339655746606	3.06691380729556	-2.48564623608447
H	0.94166812895578	3.58425821730740	-3.29654704542689
H	0.55168446202127	2.16426180206356	-2.76282521425243
O	2.51948344343365	0.15684840015346	3.24071634097570
H	2.99392564820097	0.27181828534144	4.07445954168467
H	1.62869117976903	-0.20932476369261	3.47479035846110
O	-2.63463498390967	0.09314519818085	0.84767377984222
H	-3.06678703923708	0.67939171410771	0.18647280359710
H	-2.58851310748825	0.60572065045962	1.68412211094941
O	3.84832618946882	-0.16521262884771	-1.22054988098502
H	3.21802708336851	-0.64338269900650	-1.87452954878801
H	3.54381251838066	0.81355335729440	-1.14809114262865
H	3.72807597469608	-0.60063423957221	-0.29811434983568

I-5

O	1.03347025717761	3.70615290238574	-0.27968983531453
H	0.36822652561730	3.70232523241014	-0.99701102045332
H	0.55533415438450	3.32201292558917	0.48593631477647
O	-2.97967495193912	2.04502273291043	1.93099145825096
H	-3.58665697569677	2.71835422133640	2.26643944852255
H	-3.10648134430165	2.03413776775593	0.94021022151604
O	3.44413144595284	0.92376654451322	1.47936704600403
H	2.91687843314128	1.13029689727332	2.27709714203464

H	3.15192272124712	0.02185570679422	1.22723412143948
O	-1.57459643234931	-1.34363521467519	-3.56597802498196
H	-1.84913122906903	-1.58508365634767	-4.46078884654877
H	-2.40169781381619	-1.16362768885351	-3.06613436621030
O	-3.53918268652755	-0.68179628597983	-1.74345189104081
H	-3.19764999307475	-1.13160690276268	-0.93308339325616
H	-4.46933115790378	-0.93628089051874	-1.81652439299256
O	-2.19131127396843	-1.62788336469232	0.39689841980716
H	-2.46905806822076	-1.39354369703963	1.30928934798518
H	-1.81680109576113	-2.53515408538766	0.43115477357423
O	2.88137338257988	-2.03046813462654	-2.05633899679636
H	1.97901505153004	-2.40337128707651	-2.23298417959757
H	3.50888292314031	-2.62868725206041	-2.48294775464031
O	-3.10823110607550	1.97672109099858	-0.69662653398738
H	-2.44938026558666	2.47866428437689	-1.21609814282532
H	-3.27172585663709	1.14714845886877	-1.18060878362697
O	0.05480781778910	-1.15718332523027	3.49129066086591
H	0.59959671428600	-0.35346319618127	3.60496104737055
H	-0.86099976833956	-0.84941804834543	3.36489119121508
O	2.27350067214940	-1.38798477792280	0.52509895462219
H	2.06433945171320	-2.15477078289130	1.09666129200918
H	2.64393783395136	-1.74693294328527	-0.31097282378033
O	-0.00138402003388	0.79843045069898	-2.64601802790855
H	-0.03309110873521	0.48688991140303	-1.70810034073590
H	-0.49535168225665	0.11506076263738	-3.14197120910912
O	-2.68740478522303	-0.50355713177906	2.86751831482464
H	-3.34928033698277	-0.75873326922402	3.52452699065281
H	-2.89562317071728	0.43249099239221	2.63187577015194
O	-0.01010733044119	0.02901662464616	-0.02442133783010
H	-0.76472285288101	-0.58435216358056	0.12676857379282
H	0.79584413190677	-0.50655127817267	0.17779001074554
O	1.54121149915339	1.27213896124422	3.54086804748430
H	0.87643400409592	1.76945711165026	3.00574096797776
H	1.68671202949464	1.78514241866380	4.34716409804705
O	0.38174446408359	-2.95472760031629	-2.23449880673983
H	-0.34359372576591	-2.49350592980382	-2.69445101658790
H	-0.00402640279875	-3.33626768789954	-1.42549178486340
O	-0.68823231718998	-3.92917050226545	0.28651946652661
H	-0.03453305736163	-3.82702826193128	1.01945519358803
H	-0.97651107274648	-4.85142523597380	0.31995638943472
O	-0.19355165265857	2.16969464803618	1.68410109153438
H	-1.14527936285910	2.28546147010500	1.88285318454423
H	-0.16347484034828	1.38042216149070	1.09015044215924
O	2.77394881003745	0.76490628566616	-2.53028791593661
H	2.95927496974271	-0.18922046036037	-2.41938351060800
H	1.82003057556313	0.79238407411194	-2.75693236973950
O	1.08948037512915	-3.23083004251405	2.23520245962703
H	1.50185615089086	-3.82253050640003	2.87887727160784
H	0.68183637617224	-2.49168368680420	2.76851978114312
O	-1.02744865047508	3.24691087970999	-2.17365800422896
H	-0.66809973416806	2.41180445038938	-2.55966492518085
H	-1.22009244129948	3.83281259389649	-2.91811438640369
O	3.19484683852138	2.38259585046541	-0.59644389259872
H	3.06708219316605	1.72103149187631	-1.37030911050464
H	2.33341098773890	2.93493883622400	-0.50718486846719
H	3.30849677385348	1.83514355038178	0.26669699965991

I-6

O	2.60272772225073	2.88557895806340	0.25109783232789
H	2.00568608808197	3.29263185521070	-0.40850512412155
H	1.99881745802172	2.62622465052934	0.97936109233826
O	-1.77287801778414	2.73958621285769	2.51681907395745
H	-2.08384900896645	3.42207203436135	3.12638337713935
H	-2.21205583363418	1.90402205603850	2.79355203169780
O	3.48520281390041	-0.94395561090555	1.35197448740123
H	3.09520234217366	-0.66963037465308	2.20602867534081
H	2.82707962451804	-1.56801282982351	0.97822377424611
O	-2.00344038359646	0.15313401809709	-3.55431903748334
H	-2.34825748957315	0.24745427403114	-4.45237354268228
H	-2.65475527412475	0.59877269944929	-2.96612918346743
O	-3.48092315430593	1.31264881923653	-1.54335494976112
H	-2.91809248656426	2.02680278968429	-1.12615339372006
H	-4.35602071547410	1.70820019620968	-1.65465273071986
O	-2.69685676825876	-0.55553329626940	0.31730555377838
H	-3.15030057951709	-0.00695358951525	-0.35988269422993
H	-2.77724422577334	-1.49390945297744	0.04038572098603
O	1.71658183233861	-2.66863740481117	-2.56519539516043
H	0.74548034530758	-2.57324011979550	-2.74779494303515
H	2.02520963654159	-3.39344208540493	-3.12464481538386
O	-1.96444731054768	3.07872558516910	-0.31854475516935
H	-1.11281764144629	3.41779829213068	-0.65680664714744
H	-1.90072680562146	3.05963938292616	0.65401559648303
O	-0.46678187080630	-1.68310150568179	3.26547215928398
H	0.37078423625254	-1.21357407007072	3.44926562746964
H	-1.16732122520393	-1.00625850622532	3.27357702277850
O	1.42035856864756	-2.30506082386718	0.12314369864458
H	0.88515581157097	-2.99263274456017	0.56924706452788
H	1.60032448653278	-2.62583223992228	-0.78796429996341
O	0.36580508584120	1.16734573551633	-2.43632918045643
H	0.19217401526708	0.74909178636689	-1.55766998687487
H	-0.38340810452856	0.87142143412458	-2.99160814795692
O	-2.67583131161264	0.16152315279199	2.91807652130913
H	-3.46714588708708	-0.06507280900315	3.42587543859917
H	-2.85501151086186	-0.13928797665049	1.99376283649137
O	0.00568918865211	0.03807682245058	0.03128369812760
H	-0.94152580877084	-0.20211821162547	0.14330952566464
H	0.49273853347051	-0.82170204735756	0.06585527724347
O	1.91847395502813	-0.15691676457195	3.57105800553616
H	1.55312330496278	0.67280721865999	3.17927841293446
H	2.27042784930411	0.08171615143145	4.43919499748914
O	-0.93074420960164	-2.37407669181584	-2.73942779827537
H	-1.38120610614370	-1.56275564066830	-3.03801412680632
H	-1.43775476796693	-2.70129330452521	-1.97464279480357
O	-2.35475460678039	-3.20599172551887	-0.35652304303265
H	-1.74239301111511	-3.51655709615136	0.35321073987751
H	-3.01883619191572	-3.90250878860848	-0.44895897206901
O	0.79425814102361	1.74316563435780	2.01859567876893
H	-0.00926379154974	2.22209549507919	2.30517220162768
H	0.47078639047259	1.12461969583581	1.31840742278079
O	2.84522812507373	-0.06933808737520	-2.56639452242706
H	2.59925254060501	-1.01409403059921	-2.62921410341464
H	1.99486506381799	0.40155526826663	-2.69901952932322

O	-0.48520336302026	-3.70769041958678	1.57595694867890
H	-0.38839776224572	-4.53207960230384	2.07141084995209
H	-0.51267714048458	-2.98259888135352	2.26237721011115
O	0.54141807503804	3.69575505791619	-1.51620625675758
H	0.49255283180976	2.86717149814366	-2.05224280439761
H	0.63232699375322	4.42326349869530	-2.14639510193761
O	3.93844734517368	0.81924549265019	-0.43464171175925
H	3.52996191647624	0.43521914035244	-1.29454812878274
H	3.41960560829356	1.67220319009058	-0.19194275117678
H	3.78493043468154	0.13350863547398	0.31572091870369

I-7

O	2.73497612617722	2.67311059652589	0.47604637064698
H	2.59597307225717	2.82085384458835	-0.48092513971064
H	1.83274034614098	2.74227142915554	0.85480441648613
O	-2.19276081002878	3.45264390241636	0.25349281913979
H	-2.60046150549198	4.31934236269276	0.38351071001010
H	-2.84910237306340	2.79267450563923	0.57278254673348
O	2.25185009574170	-0.38420235496390	3.13322311084218
H	1.57998968322495	0.21263697028370	3.51994375314808
H	1.72084740923190	-1.10201792683341	2.72730592516326
O	0.00944512903072	-1.16577397638518	-3.80400164508885
H	0.37440252071935	-1.41217071168380	-4.66518570101236
H	0.73128907402393	-0.67223270960321	-3.33124217405784
O	-2.05551582741250	0.59784701236982	-3.41456369149121
H	-1.36707165835604	-0.04099126140635	-3.71734056304377
H	-2.63872257618434	0.73491502146786	-4.17332279629721
O	-2.60010687753316	-0.34194619580989	-0.88696972049187
H	-2.60355919206061	0.02101715477787	-1.79921951510609
H	-3.14534351481172	0.25431461864561	-0.32787486834267
O	2.06844023606905	-3.51576925640798	-0.28927857763949
H	1.32561090385934	-3.50109013763639	-0.94910933310207
H	2.44984459623428	-4.40263509985037	-0.33178329651985
O	-0.77448072289680	2.87517661240112	-2.16405910715690
H	-1.23665019451196	2.16481181490701	-2.64413592704329
H	-1.33910505755591	3.10571128395665	-1.40377620898014
O	-2.31359591001748	-0.07433942285652	2.86009195004089
H	-1.52060378677794	0.28293307570630	3.30601193129528
H	-2.16489326756788	-1.02968926627333	2.73629764245360
O	0.71310187094616	-2.09505291536638	1.60538027433604
H	-0.09828226070390	-2.53670418434994	1.92870950810320
H	1.20314802952713	-2.75933363399697	1.07187996822029
O	1.67174295428480	0.12802791270084	-2.16146752750600
H	1.06805959471139	0.13510794500714	-1.37861252915620
H	1.87245205798476	1.07606873050238	-2.32206012266195
O	-3.67860446296542	1.27595590574733	1.05698341695747
H	-4.59937084628820	1.31741646555648	1.34911510905644
H	-3.18928812810944	0.79634340723119	1.78575150254534
O	0.00754088350683	0.01150276110544	0.00707060966611
H	-0.91495660611083	-0.15356609704162	-0.29144430842966
H	0.23666739295986	-0.76236743486541	0.57765968781005
O	0.02465207857896	1.21175381339807	3.83466605992199
H	0.05423723240527	1.82249501552448	3.05857710439487
H	-0.00673834591702	1.77071622716474	4.62293857395883
O	-0.06709810531268	-3.31548347582692	-1.87444377644315
H	-0.10865854955690	-2.64822887174156	-2.58456636007086

H	-0.92463313289922	-3.26998910346949	-1.41355361598233
O	-2.57454777128314	-2.99947495316193	-0.44636286848441
H	-2.75751665851531	-2.05452162124961	-0.66970688381778
H	-3.31888946343227	-3.50592032301397	-0.79956530591600
O	0.13483244471972	2.36854156083696	1.39885513469576
H	-0.61040941884859	2.91599115236625	1.07945829031393
H	0.04419326274966	1.52517200186892	0.89152939243899
O	3.60472412515540	-1.16468987214814	-0.62628056537317
H	3.20083839321595	-2.04520436608611	-0.48707021376435
H	3.03637453615237	-0.74483915507813	-1.30664789126611
O	-1.88149630785585	-2.84936327151186	2.22680749359370
H	-2.22341079981545	-3.53853524876755	2.81218623399931
H	-2.24377295848286	-3.04542914145644	1.33369474570807
O	1.89737380446917	2.87704693335989	-2.21978482662812
H	0.91411579266066	3.00253390629094	-2.21279129329175
H	2.24580147607598	3.50770699749819	-2.86352920130614
O	3.79343289038025	0.50014136674728	1.30150669811854
H	3.74259822726313	-0.20086026726496	0.55297711246634
H	3.40170699642973	1.37783854210560	0.94013229657137
H	3.19070985347978	0.17991540556112	2.06973016634581

I-8

O	2.70647725591733	2.80264700478591	-0.14015631482339
H	2.23971737135878	3.03275823694554	-0.96836976517049
H	1.98432527857237	2.74028555228637	0.52131277669164
O	-1.96876793471625	3.27971579945553	1.40194698243461
H	-2.30700547373630	4.09864372627861	1.78818182711496
H	-2.47015648854042	2.54964373125615	1.83171184535980
O	3.24037705847577	-0.57642050575456	2.07706160444832
H	2.74098770434930	-0.07810687524169	2.75429468221641
H	2.61618913214286	-1.27746698930148	1.79291218653475
O	-1.46778586964341	-0.76995724432504	-3.69709234648573
H	-1.69024967981734	-0.90693470569697	-4.62775060966420
H	-2.16202359325478	-0.17005378022013	-3.34033823151005
O	-3.13130568152917	0.94064744153732	-2.31413964975439
H	-2.60091996193362	1.74557665345476	-2.04059539893457
H	-3.95856080838420	1.28961994932070	-2.67292585786242
O	-2.74959069140510	-0.45349775413790	0.02454842800434
H	-3.06690893582807	0.02567668196139	-0.77296170806877
H	-3.05574847677483	0.05562391613518	0.80673537351956
O	1.91976050292206	-3.29553367947703	-1.43413886263974
H	0.99449944576436	-3.24142096180862	-1.79112994818639
H	2.26683737273713	-4.14999381486450	-1.72247914225341
O	-1.74170882412604	2.98833736155296	-1.43979490078765
H	-0.82013668252907	3.17029942817117	-1.70960924828569
H	-1.80540406222813	3.17885374732338	-0.48585825648946
O	-1.14845084675178	-0.53198600121292	3.45725516185819
H	-0.25611935361256	-0.17859040297225	3.64154747591072
H	-1.03567619596880	-1.45533269037803	3.16613665828966
O	1.29593649554418	-2.20678091593859	0.98756127744874
H	0.66439044113567	-2.73650325484909	1.51494942027048
H	1.57980597287016	-2.76961598708699	0.23338324826064
O	0.79066209335463	0.45313687616791	-2.56869071476797
H	0.47041596222149	0.27468556191754	-1.65071602775510
H	0.11146683051957	0.03736120377149	-3.13709299760043
O	-3.05072517961183	0.94000144804976	2.38412310641270

H	-3.81040520702635	0.91372228634802	2.98186301725487
H	-2.33743859895970	0.40927189719960	2.84386698073213
O	-0.00079885509650	-0.00855535147099	0.00730943057461
H	-0.96377221236316	-0.20737610207119	0.02932370885207
H	0.43911328576787	-0.81924214742907	0.36323710507497
O	1.36429058738500	0.76908148358785	3.71407418934352
H	1.11289716539545	1.46171446482391	3.05616207945126
H	1.60025583988447	1.23730193086248	4.52638965972855
O	-0.64284470106074	-3.03820749305660	-2.13780028208153
H	-0.99420391421291	-2.31955954011191	-2.69497215020024
H	-1.27581377729541	-3.13835430146949	-1.40380297088410
O	-2.48828849800435	-3.12967904282892	0.10701677105580
H	-2.76998516368274	-2.18277671425452	0.07465311460724
H	-3.29354418732587	-3.64947850937537	-0.02125291371727
O	0.59608340947365	2.17842056991383	1.54142339903849
H	-0.21673201813907	2.72232288376621	1.57212338289357
H	0.33859806074073	1.39178591802132	1.00112770064784
O	3.18559985383139	-0.81734618909786	-1.97505279589730
H	2.89161683454823	-1.73608055102612	-1.81231765141310
H	2.39477136821937	-0.38295449915018	-2.35988097479082
O	-0.89863887557872	-3.18760561807915	2.36900114742979
H	-0.99483899263416	-3.95238683426551	2.95238711510125
H	-1.54665826880980	-3.32014253254764	1.64081902601525
O	0.95238260077410	3.13903826245284	-2.34094796340023
H	0.94008337255936	2.19507456247575	-2.63265349156135
H	1.17016695153214	3.66240381559213	-3.12417878487044
O	4.02486369767968	0.61971750104792	-0.03690258795802
H	3.71943149490333	0.01225417231442	-0.80613946524541
H	3.51869504816687	1.51076683148669	-0.11840609694956
H	3.73499052183386	0.17434008923569	0.84223222743246

I-9

O	3.35294888415231	1.35341139589477	1.33476600488689
H	3.48039115749865	1.63623956945659	0.40729695825590
H	2.48732496782630	1.74327918886537	1.58056597061676
O	-0.75669527104390	4.04631847711619	0.49697071178125
H	-0.83476972833070	4.99260677000704	0.67725606530684
H	-1.66501909769083	3.67761499769486	0.57112982940213
O	1.22432544162285	-1.51368936966971	3.30860621396509
H	0.75945140465413	-0.74299642139430	3.69207613166129
H	0.56482440278034	-1.91766079746053	2.70516184186639
O	0.41739130919987	-0.71678497315361	-3.92894949260037
H	0.85657052233577	-1.01324110164496	-4.73789178028881
H	1.13688449744998	-0.58611713919517	-3.25530599486736
O	-0.96027168143977	1.61818824224350	-3.58444403022384
H	-0.47018849768673	0.81545605246886	-3.88316903325333
H	-1.28657996127820	2.04315034546445	-4.38902360741066
O	-2.28958356514441	0.80185693527223	-1.32092911444123
H	-1.98820134935500	1.09076786017959	-2.20987259838592
H	-2.78345293044249	-0.03786392932970	-1.44507276257136
O	0.65778607237760	-3.99811557953604	-0.57924103994526
H	0.12863547401002	-3.62184697780276	-1.33020476723625
H	0.67569867882941	-4.95471656680192	-0.71459668708474
O	0.74073377752757	3.05824224273472	-1.72387896417477
H	0.15519779551562	2.66435601488095	-2.39482928738975
H	0.16441362892917	3.46798656176558	-1.05326801306907

O	-2.71729342964627	0.34269499341941	2.49170234081402
H	-1.94549503940130	0.47579188584534	3.07668441656536
H	-2.82307454471387	1.16451780570912	1.97957418984303
O	-0.45933808510715	-2.34256391996146	1.28305465819290
H	-1.42348362600407	-2.46646797189299	1.40123809167654
H	-0.14368285675292	-3.09129422850297	0.73065499053417
O	2.01161577621634	-0.32134659694649	-1.82526432154014
H	1.30815511825480	-0.14296462949671	-1.15339902205225
H	2.57537011582893	0.48215712857914	-1.79958883668295
O	-3.05578514148608	2.52208338728560	0.61460094263853
H	-3.95566309643997	2.86924715748446	0.54523677645244
H	-2.92605441374260	1.94310335012951	-0.17529682955720
O	0.02954816974859	0.04366995887798	0.02405822574407
H	-0.80291266703701	0.28827261461126	-0.43854669561550
H	-0.16999889003571	-0.81618228291989	0.46903712960403
O	-0.31575054354802	0.76486392769033	3.96045646316208
H	0.08836643800267	1.39291216685123	3.31394976896596
H	-0.30303257698921	1.20661786347322	4.82018880002850
O	-0.87692753182550	-2.78192162919713	-2.39500519592326
H	-0.52751193402480	-2.09678973874215	-2.99457710958590
H	-1.73300403474942	-2.45101938752987	-2.06856448005016
O	-3.34572167235725	-1.74435714132952	-1.26573774018048
H	-3.41743080961676	-1.92065632728958	-0.29711340044273
H	-4.17352199543958	-2.06483297113209	-1.64903756194533
O	0.69316103005689	2.02017993383299	1.79780694585198
H	0.28680632726319	2.84435185680157	1.46264882538527
H	0.41843084727484	1.33096923628733	1.14496837281814
O	2.97987171107164	-2.40723207047394	-0.25907431488251
H	2.26969101808499	-3.07553159235814	-0.33828965303944
H	2.75429532585440	-1.73625935456496	-0.93820817662376
O	-3.20839684035659	-2.02963936919260	1.45401688528209
H	-3.84364444056158	-2.49695398594948	2.01318165369312
H	-3.09249960015448	-1.12988408713132	1.87150574192241
O	3.22562413676773	2.11980552416423	-1.39226229979311
H	2.36460500004693	2.59311214901565	-1.51438507195103
H	3.88896074631985	2.63951908128778	-1.86486518743231
O	3.33120657861210	-1.13308788259199	1.92635939952289
H	3.19458638665643	-1.68592152459274	1.07213437065628
H	3.37811772048434	-0.14204554230636	1.66483538916222
H	2.50292039114793	-1.26298658529979	2.52212896398220

I-10

O	3.43237459538983	-1.74022610685937	-0.17237957217514
H	3.41856225054088	-1.40549297610755	-1.09150037313250
H	3.24048242963758	-0.94932418344793	0.37485497790904
O	2.66751380384755	2.96882231842406	0.47860066667546
H	3.52437109476679	3.38142591671540	0.65378871092069
H	2.72553703926852	2.04726185401412	0.84788591882543
O	0.69258509655585	-2.61639967368392	2.69855012458541
H	1.07339243699232	-1.85525524264953	3.18054266794965
H	-0.20419061559547	-2.31335604977201	2.44332805915453
O	-1.49010208157014	0.17495606829520	-3.68906472403790
H	-1.68767332759750	-0.11984414770250	-4.58874344459410
H	-0.75309004384034	-0.40941741646822	-3.36566692950028
O	-0.43446032493933	2.60853534551166	-3.01558517411022
H	-0.84930883369864	1.80407685207079	-3.40980874637279

H	-0.57340471757324	3.31626823405416	-3.65926683435726
O	-1.24519157363478	2.43578146981748	-0.39160796411144
H	-0.99759379020146	2.68168683934192	-1.30970490569461
H	-0.78065858467959	3.05832315869578	0.20954962829611
O	-2.71902146871888	-3.01220421966850	-0.43716794918035
H	-3.00294976995076	-2.22455534356837	-0.97258804361673
H	-3.44501496755422	-3.64747484835320	-0.49231479682856
O	2.28786984612464	2.01611126311374	-2.21085182300146
H	1.39833746641550	2.26921871359278	-2.51641745063702
H	2.41645638303633	2.44831846229770	-1.34643643539719
O	-0.47993587867367	1.55430421000033	3.30771660297432
H	-0.27149466200434	2.32882423327203	2.75546592522071
H	-1.40049233324202	1.30721840135622	3.10466418789358
O	-1.60581793514993	-1.56047775384328	1.58484097440961
H	-2.24079362052251	-0.97980241717068	2.05059660636340
H	-2.13502105303961	-2.15887553539077	1.01211591223864
O	0.40730607721117	-1.19470900984452	-2.41031046966835
H	0.30802175518583	-0.74811401844254	-1.53404517974531
H	1.34742893786528	-1.04738439036401	-2.65439449137838
O	0.25325546837653	3.72487162111845	1.51186862257483
H	0.19975022688809	4.63167256842352	1.84244450378951
H	1.18460282950512	3.60515571964405	1.20787495907289
O	0.00813340365133	-0.01278256419620	0.02258587870066
H	-0.46398332598489	0.84045432124231	-0.10210757101415
H	-0.59601731401590	-0.56191969577680	0.58139079857912
O	1.52399888847290	-0.14864242633413	3.79842907573229
H	0.75139748741814	0.46939115398773	3.73327548115006
H	1.88602315997949	-0.03183535811071	4.68649588548813
O	-3.35760852118364	-0.74197207738494	-1.68720529525935
H	-2.80953283720716	-0.38492974699025	-2.41044905599888
H	-3.54707014845656	0.01065344229609	-1.09809189319342
O	-3.72614848153502	1.51322897377613	0.10867403671861
H	-2.90298430237600	2.01720220185535	-0.10344404992768
H	-4.46096698885762	2.10756585909346	-0.09574700912351
O	2.41051132373271	0.42803188510530	1.25026629473018
H	2.28112995225349	0.24962695437512	2.20735441222233
H	1.51753022932826	0.26978994597808	0.85587015290174
O	-0.05322071306426	-3.64170049914703	-1.16187965649084
H	-0.99416184283173	-3.56824669542487	-0.90379930069986
H	0.09491734660119	-2.87839781676313	-1.75937340075049
O	-3.08755922283546	0.55522747350670	2.61913318343399
H	-3.78337964561710	0.56977234512203	3.29001337003401
H	-3.48772156369142	0.94033487915993	1.80745880248442
O	3.06063837554804	-0.49785759899481	-2.68749062369164
H	2.88227861832782	0.46682993692466	-2.53792122210202
H	3.68767618444001	-0.54299776015649	-3.42112983711202
O	1.79803390412675	-3.54547914401676	0.59551458017440
H	1.03292751412283	-3.62182809625758	-0.08509059675363
H	2.46958647965568	-2.85284136833341	0.24237453840935
H	1.40106188457675	-3.17812344095828	1.46889428004401

2. Lowest-energy isomer in each class optimized with RI-MP2/aug-cc-pVDZ.

Lowest-energy isomer of Class II :

DLPNO-CCSD(T)/CBS_{est}(zpe) energy relative to most stable Class I isomer: 1.85 kcal/mol

O	1.79316022830574	0.07320303103403	-3.30579820963712
H	2.14888393534925	0.86495926039489	-2.85684280989210
H	2.20857215845194	-0.69341229565774	-2.86425792625973
O	2.71910699781837	2.32019419384303	-1.78400998664949
H	3.59883977253967	2.64952778670575	-2.01455620785136
H	2.81447976279555	1.88709125657025	-0.89955582851696
O	2.83422744496464	-2.18209920584900	-1.93545881357888
H	3.40409333299742	-2.79744945862584	-2.41640265152012
H	3.26582534834839	-2.03802448206559	-1.06119295416949
O	-0.74759552751241	0.21507515426377	-3.70779427033949
H	0.25794978063788	0.09628935340532	-3.60383838625835
H	-0.98157255587233	0.92717893027172	-3.02522468180230
H	-1.22781209568252	-0.66306848980217	-3.36517378510781
O	-0.05960738308387	0.06725573181410	0.00368621751103
H	0.85533889250278	0.36605709205755	0.20163719145144
H	0.04193155456160	-0.84208007933881	-0.36717152943807
O	2.54512052265480	0.95400820033390	0.52723327877716
H	3.07723287883710	0.13342422233025	0.62852739699127
H	2.49343077546420	1.35677764027886	1.42175882306563
O	-1.51244541518294	-0.04121433977679	2.33273641916425
H	-1.02103346269976	0.05330862781327	1.48187376488221
H	-2.06577139414822	0.76714841056149	2.38185206267865
O	0.20850715910399	-2.46128226947688	-1.05956336959765
H	1.11448917004374	-2.58496104469740	-1.40639173190952
H	0.08210207586259	-3.12660534237674	-0.34524845709809
O	-1.23319300062297	1.80766875283941	-1.72979160778244
H	-0.80801149008912	1.24064357029311	-1.03773988739174
H	-0.74552985376401	2.65640025877949	-1.68752288413755
O	3.62372133159326	-1.57901559752946	0.62344200207796
H	4.47114815754328	-1.84403624974465	1.00649634355038
H	2.92515106672669	-1.93295048464204	1.25126631060871
O	1.73093331327080	-2.33709636527290	2.26146838016395
H	1.37153261999890	-1.67500212870452	2.88162834094072
H	0.97854271642540	-2.90570374301419	1.99750816794357
O	0.63177611332889	-0.23567292581617	3.89154510099781
H	0.45279778752278	-0.45116586088514	4.81708283089227
H	-0.25783330214544	-0.16057915665536	3.44945287012626
O	1.86115419427222	2.02580574566007	2.96672226923382
H	2.36827594675236	2.52366537388660	3.62157178614457
H	1.46597111672448	1.26259438561005	3.45264396279308
O	-0.25363825523591	3.09218644007850	1.31781878783806
H	0.49603651433173	2.88508298150617	1.90366799805129
H	0.11847299856056	3.51386394351227	0.52305025908143
O	-0.37846073144745	-3.84480520458509	1.19907602706645
H	-0.51436713942348	-4.78185881484448	1.39120846751986
H	-1.27206739753401	-3.40696164387273	1.30968394286406
O	-2.77272067537571	2.39576404605783	1.99034665688529
H	-3.23115493437909	3.02543070857299	2.56165469175665
H	-1.91440967005853	2.81862359168129	1.75034546678059
O	0.51726612368907	3.93879097434652	-1.29978642757568

H	0.51776178688935	4.84075944472141	-1.64695816891319
H	1.37553426845585	3.54189426575984	-1.56576687373933
O	-2.60945834147844	-2.46706576419778	1.41637125001977
H	-2.38249705275681	-1.62109018384400	1.85067546087260
H	-3.09004086897851	-2.19915697469727	0.60888705460642
O	-3.73326698131325	1.25999227510469	-0.47307363771688
H	-3.00726925144655	1.63380294061608	-1.00481384057331
H	-3.59945063393003	1.66257635598693	0.40476135711452
O	-1.82599436768056	-1.84238325497128	-2.79778979337895
H	-1.15452221499473	-2.24721613589195	-2.20634612854505
H	-2.60602177677926	-1.68063600478770	-2.21742795992999
O	-3.81433539337619	-1.36820601855682	-0.94312717547815
H	-4.72508485154406	-1.61188815134028	-1.15843570552723
H	-3.84890082878910	-0.39657227517066	-0.72926825013570

Lowest-energy isomer of Class III:

DLPNO-CCSD(T)/CBS_{est}(zpe)) energy relative to most stable Class I isomer 1.32 kcal/mol

O	-0.40800001040002	-2.75137106795114	2.85607435796310
H	-1.12228301758622	-2.01680327069540	2.96111392849464
H	-0.53000417942792	-3.18627732171520	1.92669288181519
H	0.50856357960650	-2.30333442906875	2.86925413651186
O	-2.20098329828457	-0.94915422266467	3.01367842480136
H	-2.55608561960958	-0.79405336516422	2.11138749243308
H	-1.93878099297846	-0.05865442808386	3.32194957031125
O	-0.60195795709520	-3.76832058598303	0.53891069029124
H	0.12362096599140	-3.37524025714663	0.00767831945005
H	-1.40909206217544	-3.59608216113562	0.01186274456428
O	1.85016879627663	-1.51817319197497	2.88543974332245
H	1.66731101802608	-0.56031145360786	2.77359968240341
H	2.53319307376519	-1.70543175178788	2.21490001033915
O	-0.06120506676684	0.00426839173069	-0.02296092298932
H	0.00986425973054	0.64725388040957	-0.76917659243370
H	0.43872300974902	-0.79497443602237	-0.31753211984640
O	-2.72615585183498	-0.54839255832428	0.32645575953514
H	-1.77823727339459	-0.32397977691371	0.15954266518552
H	-3.21863128519295	0.24502597073920	0.03153720220393
O	0.09646975870866	1.88048519524312	-1.99555677203111
H	-0.75396204885547	1.87302224843356	-2.48275732078394
H	0.16979413491448	2.74507707916263	-1.54534859507732
O	1.32479550484282	-2.25861074436787	-0.72916920126896
H	1.42897200665734	-2.27992867648527	-1.70564441530055
H	2.22615972588606	-2.22395308820718	-0.34910669005098
O	1.04187295600695	1.04534520838457	2.21836377847562
H	0.63867421769495	0.70624108256745	1.37846489452867
H	1.72293959699774	1.68017096238133	1.92129275684265
O	-2.73327663726235	-2.86018179064031	-1.06540318689795
H	-3.56040369743364	-3.34071751358960	-1.20393203198858
H	-2.96034791475544	-2.05279593545752	-0.54482301296604
O	-1.37690680321361	-1.32970150367540	-3.07721163011843
H	-1.88672388751351	-1.91811210888317	-2.48888084580741
H	-1.83252943230628	-0.46906070747071	-3.06992014691611
O	-2.41695990546029	1.37213003137606	-3.01389780422567
H	-2.86198386054573	1.70875436120037	-3.80317215304731
H	-2.99517865986281	1.61683959781807	-2.25469690958131
O	-3.65457807420705	1.91692800964821	-0.62671357377504
H	-4.51676577839304	2.33151786196245	-0.48939513600201

H	-3.01917496919643	2.41714064334537	-0.04459337397609
O	-1.87310659955820	3.02947058301983	0.98137253993990
H	-1.00643303775504	3.37991601892523	0.70892520861523
H	-1.74754744968707	2.64604400140366	1.87075963167621
O	1.21096888103744	-1.82623199165685	-3.42376452482141
H	1.35674992206863	-2.37036119695506	-4.20857543203487
H	0.23632446852411	-1.63585987322009	-3.40195594692108
O	0.60297742946036	3.94484425538557	-0.18688111364688
H	0.70272876600863	4.89327813652585	-0.34608487217091
H	1.46178377536051	3.65220267501525	0.18775065230228
O	-1.27798445600816	1.69742423909962	3.44374014251654
H	-1.26738862382602	2.14181661015292	4.30208261906946
H	-0.33565639351102	1.56142252296369	3.18655630618158
O	2.46892295295293	0.59738535239761	-2.71912775499812
H	1.66177193398476	1.14157645347805	-2.62699341628500
H	2.14336537053196	-0.25609254397610	-3.06844990474797
O	2.88439955614875	2.74344617755581	0.90971606697906
H	3.57515924924865	3.24575891899366	1.36318120471820
H	3.36435857909150	2.09049030696906	0.34328851170660
O	3.67227690806752	-1.72354372328838	0.65185484996768
H	4.49509387405934	-2.22890469853285	0.69929157333029
H	3.91660635618618	-0.85234933518887	0.26547261442949
O	4.01096114581732	0.74800500061210	-0.61593363768093
H	4.89025697084001	0.94093763143662	-0.97209260437453
H	3.42952309985495	0.65637530149760	-1.43457931813922

Lowest-energy isomer of Class IV:
DLPNO-CCSD(T)/CBS$_{est}$(zpe) energy relative to most stable Class I isomer: 0.59 kcal/mol

O	-3.19364160007039	0.38117925857405	1.85628115637902
H	-3.30885778175532	1.13888243162961	1.25569112637593
H	-3.41490687773002	-0.41453315917557	1.33756029555115
O	-3.27228745793365	2.55759592127195	-0.08693901420966
H	-4.12109133688373	2.96389912955370	-0.30870983338433
H	-3.05049118858263	1.95887124797080	-0.84280707599589
O	-3.57499942756380	-2.08002119906761	0.44121204906259
H	-4.33658382375040	-2.63264265273089	0.66208279569341
H	-3.51614017769376	-2.08393271514547	-0.54227309051861
O	-1.29487532961907	0.58138688947772	3.71462449239237
H	-1.67322671290876	0.84499638404281	4.56372055991178
H	-2.06357935879078	0.45196361063274	3.09931352426570
O	-0.25322435226811	-0.01737111669098	-0.12133933122881
H	0.44456129623188	-0.05865424297506	-0.81355854830962
H	0.00630404373901	0.67289846030419	0.53386504091636
O	-2.21037216961626	0.78147151059669	-1.81572772037032
H	-1.53682368944699	0.49177791268441	-1.14699525320931
H	-2.63866422586788	-0.06224951393699	-2.07891029128644
O	1.16774334967963	-0.34883228005435	-2.43601739011488
H	2.14574916718473	-0.36720606241666	-2.44946894509557
H	0.88369335455983	0.38693656163362	-3.01205854427667
O	-0.85099990211411	-2.40558161629271	1.03857113806677
H	-1.82305036948361	-2.48103820353225	0.95941888263715
H	-0.64790556823683	-1.54302347086253	0.60015954639478
O	0.22978724894816	1.85808018085359	1.83570334402353
H	-0.29637671189716	1.56615472034719	2.61281703336212
H	-0.16941268763227	2.69936730010523	1.53511319802185
O	-2.99732147135290	-1.85550836012934	-2.23567395717458

H	-3.52676637284725	-2.20832368381605	-2.96346435597722
H	-2.06856884376915	-2.20044167382110	-2.39297432867413
O	-0.50435743380741	-2.60604496015261	-2.63250248678483
H	0.12250661494831	-1.85761011508894	-2.68949109445602
H	-0.07375191179422	-3.22029451960051	-2.00840466296521
O	3.84120515585518	-0.38444276125445	-1.77356314907801
H	4.66229477657280	-0.53297822003444	-2.26125627732802
H	3.90353411794186	0.52708634241309	-1.40562419826854
O	-0.27918079936337	1.79539200227132	-3.48175611019086
H	-0.52152196378267	2.14397840996685	-4.34931747218135
H	-1.11329367947708	1.48250473146240	-3.06025280526395
O	1.08658130386642	2.91281417202143	-1.28082449456226
H	0.63323669774262	2.68985846309742	-2.11534075716601
H	0.43224859688036	3.40444064682232	-0.75203759188458
O	0.82859368221743	-3.88516015474551	-0.47109674374656
H	0.93134138851872	-4.82579341297724	-0.27276548551903
H	0.16070793189690	-3.53714147101063	0.16841084160626
O	3.55391239834514	2.10709335002145	-0.63682069976022
H	4.17039691833287	2.84116645495503	-0.76398366210773
H	2.66680505652062	2.46378044784779	-0.90457258306356
O	-0.94392909731464	3.95718052194568	0.45822170049594
H	-1.07085622721695	4.88999781563872	0.67621813698050
H	-1.83944572653982	3.60329133289787	0.25467809784083
O	3.01229289628058	-2.25677447801091	0.21243655083701
H	3.31995235770923	-1.69400166117474	-0.52305091178498
H	2.30779010644847	-2.84510367829881	-0.12915086386538
O	2.91833325121616	1.35488076832505	2.01817885470751
H	1.99185109386740	1.68211975715385	2.01233797701652
H	3.27150287945209	1.61815501184542	1.14481261700413
O	0.43299245281955	-1.61824469614158	3.35508871345436
H	-0.12456360984346	-0.86873263442788	3.64743422536109
H	-0.09447657616011	-2.02557997932876	2.63385519959842
O	2.78073187393091	-1.13628891473940	2.50151298770808
H	2.86495584394715	-1.61131567026244	1.60237781260251
H	2.87149229472438	-0.12716607332031	2.31358377249287
H	1.83586031273617	-1.31311039714727	2.87410405904261

Lowest-energy isomer of Class V:
DLPNO-CCSD(T)/CBS_{est}(zpe) energy relative to most stable Class I isomer: 1.87 kcal/mol

O	3.49132219915552	0.79535552374821	-1.66858492092017
H	3.16775952932881	1.66952177389219	-1.29936423759476
H	3.58895463588288	0.10216854164861	-0.89955895314818
H	2.79987739974124	0.43575721134023	-2.36648172839731
O	2.61873732838629	3.04307185587732	-0.58705907519060
H	3.17789791088929	3.82651741546524	-0.68248860554358
H	1.69184284768406	3.32638092063298	-0.86260836281594
O	3.68533323521604	-0.98076758809086	0.10679979168263
H	2.95667971171845	-1.62372700968828	-0.03530103006933
H	3.63032544119109	-0.75910914923905	1.06006542180868
O	1.80216323497688	-0.02457338468488	-3.33645076017330
H	0.94546054745319	0.43500731189257	-3.18021254220369
H	1.55534774483783	-0.97262716085884	-3.32859437754738
O	-0.00941010738830	-0.03000443820006	0.04686815025274
H	0.57898195478000	0.53358621299456	0.60498259251944
H	0.48828977000412	-0.87246569617389	-0.08721662173472
O	1.59453053488623	1.60473535893563	1.57149545914687

H	2.11369018978281	2.20008541850603	0.99594825281653
H	2.23982491719963	1.07588177351921	2.08880628574076
O	-2.43629039857811	-0.53154908395812	1.10417093413185
H	-1.53909996894427	-0.32772812133977	0.72851676093324
H	-3.02035485358459	0.14373418124295	0.69965117111277
O	1.36053912136317	-2.36796880593968	-0.36853601409608
H	0.97015293067647	-3.05408678999360	0.21065104094207
H	1.21029931641080	-2.65909112422320	-1.29459126759231
O	-0.48682767226530	1.12709336893258	-2.39458602079629
H	-0.37680573469666	0.68185234800346	-1.52153182856657
H	-1.39713592365027	0.90324570196305	-2.67081427801935
O	3.08052367781032	-0.36356855532948	2.76004273871875
H	3.66173310128782	-0.41319450480577	3.53018470240667
H	2.28261707441948	-0.91387425162793	2.97976863819262
O	0.79992060355300	-1.65921600012400	3.18471211863189
H	0.01113606380719	-1.13597090888058	3.42459961064950
H	0.47024486676451	-2.43501841435557	2.69723945854504
O	-1.54387454572518	-0.09022091789149	3.64799477239001
H	-2.10797665110253	-0.28509115702957	4.40825307283673
H	-2.07159729410290	-0.29932243080252	2.84403279679219
O	-0.77747973307845	2.51503963558969	2.71660920921208
H	0.13282087155050	2.28590309233577	2.43970190364848
H	-1.09395456782112	1.71700102567774	3.17910511498183
O	-2.02177923776687	3.46691288134190	0.60462436222254
H	-2.35208214892726	4.34518240597791	0.84047019060921
H	-1.59947228490687	3.11321755190710	1.44284380385581
O	-0.08960767358863	-3.82946609941488	1.48739024644462
H	-0.01164582714910	-4.74404840199213	1.79041537156791
H	-1.00608457645783	-3.73594962804359	1.14403350313965
O	-3.77872886305237	1.56125347975504	-0.21826713354004
H	-4.67470755269678	1.83382920694896	0.02297311863246
H	-3.19830623104548	2.32442207212545	0.03764391794121
O	0.16118395438175	3.57844392189374	-1.28457799065408
H	-0.13821231337641	2.82274743439678	-1.83240865799518
H	-0.53392257771690	3.65408699817629	-0.60304190603955
O	-2.51704151647816	-3.11862266600119	0.34791730804074
H	-3.35099105616961	-3.58841418294556	0.48421542355276
H	-2.66123776105904	-2.19720002700134	0.67967494453680
O	-3.16369719610896	0.31153531512289	-2.64019151733937
H	-3.77168522372434	0.47283070728800	-3.37420727498154
H	-3.53227421187380	0.80112862779417	-1.87399924041562
O	0.71120906913933	-2.60124895730521	-3.01464177276167
H	0.78637543540608	-3.30667453218177	-3.67094187639342
H	-0.26130795828939	-2.46754287929554	-2.86690681559746
O	-1.84342523067440	-2.24139097447293	-2.32595869154922
H	-2.10751074293127	-2.61922670160936	-1.46720527111555
H	-2.37957658475363	-1.43637073142565	-2.44038941584483

Lowest-energy isomer of Class VI:
(DLPNO-CCSD(T)/CBSest(zpe) energy relative to most stable Class I isomer: 3.61 kcal/mol

O	0.49178519039072	1.04253188826021	3.49158956871957
H	-0.33235875526201	1.48869641354606	3.21375496630885
H	0.60266884291587	-0.42140017867916	3.45068002082329
H	1.24142803967889	1.54297220719612	3.09157584858102
O	-2.01904020922031	2.11101011481922	2.67299263302068
H	-2.55179403038566	2.51078609807114	3.37411411500056

H	-2.00098895091095	2.77438101717991	1.94003861212865
O	0.74163882469074	-1.45264948139947	3.37057131692383
H	1.28861190352680	-1.59671087612853	2.49713984044661
H	-0.14942090524900	-1.88430897576805	3.22158823852207
O	2.51962443626182	2.34271293820973	2.18088538810949
H	3.11795010471765	2.95209980005419	2.63320907218036
H	3.10396422363875	1.70691840874308	1.67058088897415
O	0.07562174366584	-0.05462875516434	-0.03169465680149
H	-0.78050416963734	-0.16514342191968	0.43546302305286
H	-0.10953981955136	-0.33986327761377	-0.96424673924900
O	-2.34964544999203	-0.31033420152696	1.39879079495139
H	-2.41676758097838	0.53909566675526	1.88681779470949
H	-3.05447001865055	-0.29264904031677	0.71117700577074
O	-0.41186030397694	-0.89298780552443	-2.55471048175081
H	-1.20339576346201	-1.46597755954143	-2.55684468942555
H	-0.61406636234933	-0.11631672613256	-3.12222168185248
O	1.95574677140935	-1.57757520415283	1.17158297358436
H	1.30920073154597	-1.05438044264679	0.62924399587589
H	2.02990261818183	-2.44416664441611	0.70565634251770
O	0.71395970408765	2.61859327791764	0.10524704109771
H	1.38214786718595	2.71470867117583	0.81689519832289
H	0.52877299380941	1.65003607936058	0.06637564255423
O	-1.58344668957573	-2.50443248589652	2.67907872386119
H	-2.18830365307144	-2.92749652003030	3.30410127005982
H	-2.06193653715004	-1.72484513502259	2.29550482006429
O	-0.87952305940321	-3.61411086401484	0.01867635680322
H	-1.18699091234035	-3.39463783119215	0.91331182955738
H	-1.55490067404811	-3.26980961879579	-0.59543838903109
O	-2.64460546338303	-2.45193056742686	-1.90243578329985
H	-3.17797463775814	-3.03570001397924	-2.45861261757969
H	-3.25722545265172	-1.75132808589438	-1.58237884494591
O	-3.93647863020945	-0.26922393690267	-0.82965009418662
H	-4.88920174420065	-0.11358309913149	-0.88337139720658
H	-3.50780819407508	0.54816549266230	-1.22438610420849
O	-2.71662320492241	1.86824124536276	-1.70452970630035
H	-2.04004837291014	1.87140091632476	-2.41377876534764
H	-2.43081783696982	2.52606906178928	-1.04411866330252
O	1.76517560722558	-3.77766452259082	-0.40179625797282
H	2.11019331685285	-4.67857236343337	-0.35139593661514
H	0.78255974028940	-3.85071174724484	-0.31697791791336
O	-0.72551606443508	1.58886254056291	-3.64277784950327
H	-0.82629434903434	1.90957608504981	-4.54856222524315
H	0.16071045137639	1.93184086191617	-3.32682084204429
O	-1.73524062583277	3.58007405264399	0.40240106371351
H	-1.83070741245009	4.53672008302352	0.29863131609011
H	-0.76647256987083	3.38189575073467	0.28295871995739
O	2.24715997887497	-1.79879356560898	-2.45484637591237
H	1.31155859254281	-1.59161876799196	-2.64231974402374
H	2.21043588281709	-2.56590581884800	-1.85646335406821
O	1.55268532417101	2.40369380692156	-2.59824279477033
H	1.37484530332277	2.67015120776947	-1.67666969871892
H	2.29014834190900	1.76478072856324	-2.51765203116253
O	3.83184941226251	0.56312663247388	0.76896299356302
H	3.42442642811855	-0.30275152455312	0.94800232535139
H	3.82634934561720	0.62573222802968	-0.20931014522115
O	3.56361437190668	0.49044953896253	-2.04020657177295

H	4.36859939039306	0.48594571832922	-2.57569849215365
H	3.14583092053121	-0.39835147291989	-2.19339088961373

Lowest-energy isomer of Class VII:

DLPNO-CCSD(T)/CBSest(zpe) energy relative to most table Class I isomer: 2.90 kcal/mol

O	-2.23249266370544	0.87908869614864	-2.94523709698894
H	-2.89289678011743	0.62146489848038	-2.27042456920037
H	-1.88458984838841	1.75573275302321	-2.67359350164364
O	-3.97833703447068	0.03897869317212	-0.84753120599743
H	-4.86769203247000	0.41848265799599	-0.87497231680268
H	-3.52362824166698	0.46750586754480	-0.07371029864943
O	-1.03882618724621	3.26141519332910	-2.12243428621487
H	-1.21377796547086	4.06724093628854	-2.62664465394147
H	-1.22046817851668	3.48280045206636	-1.16244091370952
O	-0.68211585191555	-1.03277417162312	-3.56831758156506
H	-1.28642343161759	-0.21991844925754	-3.37799532553577
H	-0.80269994887361	-1.62274817316541	-2.74998332166659
H	0.30854184321578	-0.72613834714422	-3.53008109370939
O	-0.07306635024874	-0.05098290610691	0.03813675838082
H	0.58230063741689	-0.30265733501158	0.73333897543849
H	0.38038244983651	0.62248940658971	-0.51932718622405
O	-2.38047670093381	1.02246557010378	1.04989176135904
H	-1.53853029105843	0.59686029082578	0.75631012869848
H	-2.45628803037638	0.76879771673550	1.99509547196648
O	1.76698459004625	-0.73468349135935	1.94853945834996
H	1.65240766726033	-1.68993849736198	2.14689657630327
H	2.66698806448466	-0.62627625428672	1.58631069754693
O	1.19211814411132	1.80215237909657	-1.58567719385110
H	0.53352060653653	2.47965855940757	-1.85868984772023
H	1.89472307967808	2.26111372231724	-1.08489225594075
O	-0.85276475443453	-2.25824854207633	-1.30865970463975
H	-0.59582817225669	-1.48026773927117	-0.74830381042554
H	-1.73765776461676	-2.52528699189501	-0.97852063435507
O	-1.37706951855492	3.57729158869529	0.46653059925396
H	-1.82699499091574	2.76963601120517	0.78978817589415
H	-0.53258231191386	3.59152481987988	0.95465728112597
O	1.11307598985104	3.38375928059615	1.92218586133680
H	1.37584956589242	4.15915224074625	2.43752481624195
H	1.91222957559903	3.13581383884874	1.41374817294326
O	0.42195824319624	1.09891201081794	3.59531473846787
H	1.00406520021673	0.44737444698489	3.15775597477013
H	0.59130327841282	1.92394521820145	3.10548403943541
O	-2.02821054346681	0.07043790549380	3.61707073986366
H	-2.47695374450483	0.16716821648110	4.46676947178719
H	-1.13261655395985	0.49338435205616	3.72364384144823
O	-1.47263466529891	-2.17578506124113	2.02798065828818
H	-1.75955298206617	-1.51335568653920	2.68738801560715
H	-2.24260654337324	-2.34761731117154	1.45829552609559
O	3.21366403425439	2.53082278434944	0.20067200127906
H	3.95250953862137	3.11673974770790	-0.01419966229760
H	3.61562528446659	1.63835347921037	0.30766801211255
O	0.92466350961299	-3.32768324698623	2.19853460207995
H	0.99898087713660	-3.99029518852771	2.89747489976869
H	-0.02560951162555	-3.04697524920769	2.18568062063125
O	-3.34116026024752	-2.55745054012146	-0.13624886658031
H	-4.01405448931960	-3.24401871866547	-0.23519859240962

H	-3.76556013829827	-1.71648702209190	-0.41991825382306
O	4.07456281954280	-0.09959011961420	0.44875723675762
H	4.97499233231743	-0.30319066330139	0.73735384970559
H	3.90359834039342	-0.69879235465023	-0.32147034848567
O	1.58649971776190	-3.48765905526344	-0.55741233313987
H	0.69155034424818	-3.27193459396722	-0.88320534487783
H	1.45964830912624	-3.59050712052155	0.40570581785898
O	1.71361345464272	-0.24725561527394	-3.33053589488587
H	1.65714095745074	0.57197779743672	-2.79126601168880
H	2.29489528971139	-0.83670887578032	-2.80590826025778
O	3.31697171448142	-1.78183816009306	-1.56338059972207
H	3.98318085217137	-2.31241537318061	-2.02316326031954
H	2.69281817023587	-2.45275267707893	-1.15275255352700

Lowest-energy isomer of Class VIII:
DLPNO-CCSD(T)/CBSest(zpe) energy relative to most stable Class I isomer: 2.63 kcal/mol

O	-0.65855049466065	1.02608868283081	3.74185296817495
H	-0.85975906257721	1.83632928802696	3.12893955304207
H	-1.27369513590434	0.25911740378607	3.45713567331863
H	0.30601053124086	0.73219545213186	3.57696499250053
O	-1.22894050027251	2.94182454123381	2.18702958215096
H	-1.75678220363365	2.55460747173758	1.45558374925484
H	-0.49006105383289	3.40639894889258	1.74280708881523
O	-2.07924547359890	-0.96162605524624	2.97858394647616
H	-1.46159522810107	-1.54977364121989	2.49304827009093
H	-2.83586628550024	-0.85505945680496	2.37092432202050
O	1.77505914262718	0.36916188137772	3.24641828098310
H	2.04921767300578	0.83247642726431	2.42450964474047
H	2.02139180807297	-0.56047552561111	3.08537895556235
O	-0.02607762515147	0.13062452341221	-0.04206931327760
H	-0.89105839022338	0.59863846514901	0.04624006228767
H	-0.11045655324575	-0.69977744333527	0.48105476819159
O	-2.41129533429991	1.47230947318010	0.17633851133814
H	-3.13316277153530	0.84412835163529	0.38271216234681
H	-2.60548729241678	1.84210888969432	-0.71307335275375
O	0.47080806067456	-0.52145511960680	-2.65086097443251
H	0.30453871810245	-0.20710524463331	-1.73072126248749
H	1.10649835417661	0.12522248664985	-3.01166130954970
O	-0.19459565855698	-2.20620228713926	1.41270548593120
H	-0.45041135733834	-2.94149415054538	0.81679502774097
H	0.68429600958593	-2.42790227902383	1.78451046458806
O	2.13897440848001	1.54102298650321	0.77082338814137
H	1.35321207213975	1.05053581565826	0.42055724396646
H	2.88675732083783	1.23892090320864	0.21796127324337
O	-4.00256957914776	-0.69611600068380	0.86639260850484
H	-4.95395035070308	-0.74598785568934	1.03052642086251
H	-3.82511558500282	-1.31578730052604	0.12055610310191
O	-3.21791404754211	-2.21553323005503	-1.31770083969872
H	-3.91574375707618	-2.67641720449114	-1.80554361921759
H	-2.88196209580805	-1.52366634488644	-1.96975907381376
O	-2.28885624340577	-0.46606057713626	-3.02351189492652
H	-1.31406725881619	-0.52103301760569	-3.10185192259599
H	-2.45747956176248	0.49046552769994	-2.92348320614344
O	-2.37152584904333	2.31867036619780	-2.41784557280074
H	-2.81384651249942	3.02917373153020	-2.90032235756578
H	-1.42152368493006	2.58950461619503	-2.34651856826871

O	0.17716439742757	2.93405939444032	-1.90046241650853
H	1.00911386767226	2.56947423125715	-2.25091521187296
H	0.41847379568685	3.44342565991703	-1.10511672778116
O	-1.07018825448139	-3.84015388977406	-0.61776115381346
H	-1.33926849771425	-4.76845640531961	-0.61753795876641
H	-1.85938521593208	-3.33712221066990	-0.92791254218336
O	2.42285075051716	1.50112213063303	-3.00598079415729
H	2.88108622435470	1.80525453446507	-3.80077914689207
H	3.11998117759038	1.15234286485595	-2.41305000141227
O	0.92087678928133	3.95878525877657	0.67550256551867
H	1.39417310040594	4.77494936966463	0.88529774417472
H	1.56957524633462	3.22335618926430	0.77092498737001
O	1.45557924046552	-2.92757396656257	-1.74064529109830
H	1.12955246178601	-2.12933040894829	-2.21250307180606
H	0.64652148303155	-3.36025636582222	-1.41389660402531
O	4.03625652400738	0.38703222885000	-0.98039433569980
H	4.98572953873797	0.54954015563707	-0.89564436163171
H	3.91597041758917	-0.57070426351786	-0.77435069853654
O	2.41202364084653	-2.30403707055761	2.32502516515748
H	2.84523322431765	-2.95237385017516	2.89655154923828
H	2.92030857498409	-2.30692647144089	1.48177481795543
O	3.48622126520967	-2.23246942182487	-0.24343492718018
H	4.18507428148513	-2.86743562650553	-0.45672920270650
H	2.69898981403899	-2.53648156639830	-0.79295266318601

Lowest-energy isomer of Class IX:

DLPNO-CCSD(T)/CBSest(zpe) energy relative to most stable Class I isomer: 3.53 kcal/mol

O	0.24937994612122	1.69945838019139	3.43569375652928
H	-0.57070324581484	1.05297033311387	3.38803175785586
H	1.09510795853009	1.17696694001894	3.31402361175778
H	0.16649934621511	2.41326353482724	2.67954096619877
O	-1.68250025317515	0.09518650729025	3.31807606667996
H	-1.36784314022438	-0.72763962956460	2.87192947565534
H	-2.48284844936860	0.33892133734990	2.81406741366822
O	2.51197770922237	0.36990692662038	3.03201278803699
H	3.15126931783514	0.42789809706986	3.75598092413423
H	2.39229234001375	-0.61727603069121	2.84539760185419
O	-0.07370903722597	3.40178779285912	1.61594824460186
H	-0.80866029113512	3.07405668629408	1.05051691309393
H	0.65630486227658	3.59789147870649	0.99270687447054
O	0.01496654978175	0.07107175820328	-0.04845675623620
H	0.89599643170196	0.48050431332236	0.09504966251518
H	-0.64268167321351	0.80572862999455	-0.00322131978559
O	-0.59982192871156	-1.86105450367281	1.79599101880644
H	-0.38710660400963	-1.23490724062618	1.06476482833667
H	-1.14791846634954	-2.55900599936352	1.38079520034898
O	-0.13777739214889	-1.10877503719864	-2.52140080770386
H	-0.04185564166232	-0.62482586601928	-1.66835107534812
H	0.56568740539292	-0.73209742397317	-3.08220016865027
O	2.60505103345321	1.04190024390412	0.34088201813034
H	2.80370358884141	0.84742627785457	1.28045916115956
H	3.15300450318751	0.42851492815153	-0.19432701932517
O	-1.89569874950525	2.03142860059702	0.11393864770543
H	-2.69462976849708	1.65146277478656	0.53494435193377
H	-2.14722339576285	2.29182150079045	-0.80024184065648
O	2.07976208288005	-2.11158252134965	2.41512267481678

H	1.10929393218775	-2.20688708186324	2.30747340691938
H	2.43735502716673	-2.45631840300826	1.57372263946819
O	2.73514302404816	-3.04638227767599	-0.21318049874849
H	3.21973183031285	-3.88158613982034	-0.27295004105472
H	1.82248989481090	-3.26103266861467	-0.57137589766768
O	0.37241070773781	-3.52501131873443	-1.27025161656904
H	0.16530490498214	-2.76822970001151	-1.85911235785073
H	-0.45345870774762	-3.67915966277276	-0.77889988893434
O	-2.14221043945490	-3.54738564314580	0.23323086963800
H	-2.60414521902225	-4.36629422842345	0.45647042066088
H	-2.84017357576030	-2.91715535638537	-0.05612168956884
O	-3.90494945424983	-1.48227128688309	-0.44647397486053
H	-4.77982193684869	-1.77356303821918	-0.74074231794285
H	-3.50988503929707	-1.03272308174942	-1.25568581866586
O	3.75458398504034	-0.81563542958225	-1.37479709357717
H	4.70776274955086	-0.92900257347742	-1.49327371761214
H	3.43187637375930	-1.68527053919597	-1.02104466966543
O	-2.81502073288369	-0.36280163634126	-2.54991848169957
H	-1.91413204787034	-0.70059871887750	-2.73445459597830
H	-2.71671486577951	0.60403874197681	-2.64099982138635
O	-3.81384864290717	0.54758797841022	1.44831083258443
H	-4.70292252535759	0.81279004667014	1.72031111051270
H	-3.94090563594417	-0.22174913669218	0.84414124120385
O	2.17460144605121	0.24872572716465	-3.45519641075655
H	2.60007802845823	0.25036266259962	-4.32311210492086
H	2.83017141688013	-0.14156733178377	-2.83955986401973
O	-2.10594645703770	2.40487949294585	-2.56890510804566
H	-2.47260371706189	3.10245428039437	-3.12772794103918
H	-1.12310244521243	2.44960873022442	-2.69308407653741
O	1.94144042253591	3.57728445198038	-0.32494224377047
H	2.60613345907357	4.27615084128433	-0.38875818743482
H	2.42235002983556	2.75043836036284	-0.09394560177878
O	0.56977941604463	2.49295704610340	-2.62353999147335
H	1.18148892458348	1.80058113762112	-2.93217753344643
H	1.03877983072722	2.96744796603284	-1.91344394656655

Lowest-energy isomer of Class X:

DLPNO-CCSD(T)/CBSest(zpe) energy relative to most stable Class I isomer: 3.43 kcal/mol

O	-3.84675946638026	1.48430564231089	0.01314367322911
H	-3.27758728586405	1.79023192294737	-0.78727262423378
H	-3.94888033354393	0.46311091377701	-0.05125249833303
H	-3.31096246403239	1.69532177321303	0.85980470265948
O	-2.38988367844472	2.16849821289768	-1.96034420303078
H	-1.88924731758598	1.38164780637157	-2.26657081345100
H	-1.70223727876334	2.83851586215384	-1.77297235585798
O	-4.05908104025821	-1.04421411638809	-0.15507195914894
H	-3.76195687981062	-1.57479333852528	0.61292638478763
H	-3.65112193126827	-1.47555601853595	-0.93426632055640
O	-2.44454707789242	1.98022952343172	2.09820051041285
H	-1.72203859036599	2.61437344656273	1.92161436680504
H	-2.00466319854079	1.16072868988779	2.40987985007505
O	0.06125093646446	-0.19031321533582	0.13102241640947
H	-0.31850760142876	-0.07916339715108	-0.77360369224430
H	0.65844465551732	0.56767763574271	0.29023003312576
O	-0.95260255824458	-0.15520755775830	-2.39481014825193
H	-1.50176434868668	-0.96583908498925	-2.49779118811443

H	-0.17944157263266	-0.25371643778640	-2.98554739164667
O	4.27345330109317	-0.75360635474895	0.00539373245483
H	3.92829721036601	-1.23661117970071	-0.76777765056798
H	3.93087158852546	-1.23973251673347	0.77893689958718
O	-2.88113733488436	-2.52374204744695	1.92344986715461
H	-3.37205554239281	-2.95416463149526	2.63584256928090
H	-2.29152714937327	-1.86194530949709	2.35262952299887
O	-0.27296788798463	3.71972001126798	1.42773561112453
H	-0.26396051800565	4.58006317804140	1.86923507733172
H	-0.27650356653076	3.91560533092511	0.46135736209040
O	-2.57886725837255	-2.33200306292181	-2.16991857198876
H	-2.98941986568361	-2.89951465361521	-2.83566804311242
H	-2.11995949480387	-2.94041547459245	-1.52379300358562
O	1.20913657354063	-2.64605428997254	0.02994119933987
H	1.78017448837627	-2.71514131245053	0.82123895760956
H	0.83790691099762	-1.73211633133826	0.09758729884447
O	2.83311557554238	-2.18165225925102	-2.07529918278463
H	3.18734702349273	-2.98115448436426	-2.48805506468823
H	2.21195563435818	-2.49563107644868	-1.36801838362394
O	1.59479710900405	-0.19150693768713	-3.53076440563542
H	1.88036145583014	-0.16019438279278	-4.45348489608220
H	2.08350347965541	-0.94368055225178	-3.12226256952226
O	1.60936949744696	1.90808478509024	-1.63297930827392
H	1.70336018923616	1.29411860838476	-2.38640963955462
H	2.49498511456349	1.97607466265066	-1.22274079823455
O	-1.40996239581217	-3.71130194869099	-0.24457513294871
H	-1.86199520071225	-3.47616207217996	0.58713681787169
H	-0.46709301100778	-3.50838257854306	-0.08958567106053
O	3.85334904896490	1.86162746981284	0.01335494135395
H	4.68314424216578	2.35509283958940	0.04124595378395
H	4.10295939065728	0.89680029756613	0.01102289028620
O	-0.18882155922800	3.82830350420392	-1.31484573434340
H	-0.02778829043194	4.61555097132334	-1.85242489945904
H	0.54290539114195	3.19135090279683	-1.53427497568384
O	2.97874451079972	-2.15481333903556	2.16766318278892
H	3.46166784708951	-2.76308508444384	2.74301995294941
H	2.51841257324546	-1.52677167920299	2.76915301203213
O	1.61153404728536	1.66308938624410	1.58661919340001
H	1.07545703330946	2.48183793669314	1.62418472458282
H	2.46235731500836	1.90336141509509	1.16023044089850
O	-1.17546014730849	-0.47938320611665	2.52325800928128
H	-0.38603367239225	-0.50723551779273	3.09707234913464
H	-0.78621693273251	-0.40504697095951	1.61836098221795
O	1.51662455897949	-0.21248034534963	3.50763662740142
H	1.77186786954405	0.08812474523332	4.39057810640088
H	1.62587891919882	0.57399317187939	2.91475718631426

Lowest-energy isomer of Class XI:

DLPNO-CCSD(T)/CBSest(zpe) energy relative to most stable Class I isomer: 3.98 kcal/mol

O	0.68789187375654	-3.58723088408429	1.26974939953521
H	1.45480678030451	-2.96757751242530	1.56550777305698
H	0.62460463825366	-3.51353290234057	0.25374826944042
H	-0.18655804872148	-3.22995376063165	1.68463935353551
O	2.77984346358629	-1.81852677918829	-2.27731991970202
H	3.31363462228692	-2.17536908067521	-3.00003170221853
H	3.41350472910726	-1.63707071894948	-1.54252829691552

O	3.81652634494588	1.51499402166508	0.09205830516672
H	3.37290090156165	1.91643103471016	0.86146611987721
H	3.43082296534038	1.98162421635595	-0.67205397093992
O	2.52362822536963	-1.98228753109750	1.98923330125681
H	3.18102147008769	-1.74675921617419	1.30321240983375
H	2.08961059377418	-1.13650846116959	2.23058048222714
O	-0.38736816449784	0.13869725332762	0.00282462802896
H	0.21286695704657	0.12228349633063	-0.77994423722351
H	0.18163175247625	0.11626543131646	0.80823221624548
O	1.03826712388392	0.34057791192608	-2.29101492991608
H	1.71434051949583	-0.35093459360555	-2.44727994889599
H	1.50434381784855	1.20035723612677	-2.29839995480637
O	-4.56473745037500	0.40130922432735	-0.01945613940775
H	-5.51978533897204	0.27709067302726	-0.09769964984568
H	-4.29790025780896	0.94339896933361	-0.79643444471002
O	2.12414990811850	2.79963589304094	2.03806377702753
H	2.45526001120221	3.38675225350765	2.73105687296666
H	1.59857976841194	3.37148734295265	1.43559009163420
O	4.23604043325014	-1.09777547362404	-0.07671382419944
H	5.18257989410067	-1.25859911472470	0.03912964202861
H	4.12695700162262	-0.10962243440712	-0.00667666375821
O	2.25902682725739	2.84764974683564	-1.90069596172524
H	2.57875606908534	3.43001159303485	-2.60281030703457
H	1.68866401462879	3.40979200518189	-1.32980202725530
O	-1.52769278649990	2.58403848181435	0.00711368183037
H	-2.16681661784104	2.55355477366839	-0.73533879983173
H	-1.10941001622707	1.68578505917112	-0.01974081898817
O	-3.29336500641759	1.79922284795260	-2.03053176016227
H	-3.72570126723361	2.32579837422638	-2.71610550713468
H	-2.69682162480557	1.16615314091084	-2.52298961872557
O	-1.63091119540232	0.11947969723744	-3.23572483314275
H	-0.67809132200143	0.24450631165187	-3.06365144857647
H	-1.76768903334076	-0.84035486953544	-3.12466100370796
O	-1.82905148526855	-2.61004064404143	-2.40164996826300
H	-2.37050176565059	-3.30991141246678	-2.79114101931713
H	-2.27508000052498	-2.34993863905355	-1.55848637457378
O	0.66805240002750	4.07378048398212	0.02277233944452
H	0.47884670333143	5.02179072798167	0.05015426213251
H	-0.22343529347299	3.62565609449271	-0.01446465261064
O	-2.55710291155224	-1.53112996060554	-0.04213065525542
H	-3.35547293074508	-0.96306519127697	-0.01127055049920
H	-1.79918408460910	-0.89915338706940	-0.03931026241095
O	0.62299735087134	-3.38536441818943	-1.28285650705526
H	1.35617830064152	-2.89357723972459	-1.69978420878859
H	-0.19730798799133	-3.14458072098014	-1.76117194756417
O	-2.99061051321059	1.41133935929557	2.20231264717161
H	-3.69379276410039	1.04466012435116	1.63695541295436
H	-2.50585815419924	1.99709678364073	1.59090500703696
O	-1.48775751819566	-2.68978831700870	2.23033744080712
H	-2.03350522408316	-2.37236999492735	1.47853073947378
H	-1.44731616863621	-1.91875929552507	2.83216673925118
O	0.97017959817636	0.27692617746998	2.36326632174994
H	1.32413819239574	1.18480178944785	2.44822423582424
H	0.21799986003765	0.19766474939732	2.99207872933263
O	-1.40852682929751	-0.21423255248226	3.61773271155993
H	-1.65752690111281	-0.14973548542250	4.54950456312423

H	-2.04548536948979	0.37729571171395	3.13184780160731

Lowest-energy isomer of Class XII:

DLPNO-CCSD(T)/CBSest(zpe) energy relative to most stable Class I isomer: 1.37 kcal/mol

O	1.02720526798579	0.82866195635536	-2.09575067699260
H	1.72411815286317	1.52930361153758	-1.72174353216218
H	0.48019408851470	0.54779084734117	-1.26477356501723
H	1.52180881492031	-0.05083389438089	-2.39548378725939
O	-1.49026326632012	3.20521327256759	-1.16011863318852
H	-2.00317432552633	2.55706790020005	-0.63734592091749
H	-1.50804774249192	2.86901891880759	-2.07243158704217
O	0.09633995402413	2.72501034818608	2.64363697158700
H	0.25502608290688	3.28709120242725	1.86175511235750
H	0.75285117123494	2.00762215715290	2.55083943982392
O	2.59547898315605	2.48670862588518	-1.09694865302347
H	2.04019611611993	3.11302443465709	-0.57409589492015
H	3.18629473286115	2.01691210996963	-0.47443493309479
O	0.68605802685787	4.01202783770040	0.14259757353664
H	0.73793478314267	4.97745644818715	0.12113192210728
H	-0.12265963784452	3.77894432111069	-0.38916478974180
O	-2.54682039690283	1.16597581870022	0.43716212813065
H	-2.68104593985176	1.35068543575779	1.39344331150093
H	-3.25666910994825	0.55879460225150	0.14853352947245
O	-0.52671539696718	-2.69143218061637	-0.17464463894874
H	-0.43775441672956	-1.71345686113155	-0.08603864513326
H	-1.37290942943898	-2.88625211713935	0.27688710804994
O	1.64910323737267	0.47947555124907	2.00434658115305
H	1.63950772854926	-0.27183770027618	2.63738765833909
H	2.56194400533427	0.54003775708664	1.65808673534869
O	-0.14036922560258	0.03837274206519	0.01138716833250
H	-1.02041518341077	0.45023203332821	0.21835670317056
H	0.45409538141196	0.20742949821536	0.78691956154940
O	-2.24723199838085	1.58018267749294	3.10561563223424
H	-2.74851661019732	2.03036243145703	3.79774857596074
H	-1.40020143823485	2.09616307624120	2.99703669772174
O	-1.37834328249902	-1.10233990129065	2.94600172556638
H	-1.75975117459440	-0.22407690656884	3.13990273010145
H	-2.04487944616395	-1.59093866402726	2.43125487187196
O	-3.00853321525175	-2.62431161677661	1.13647681111513
H	-3.59469396515140	-3.33134549887269	1.43719007067069
H	-3.52436454356132	-2.10674887185247	0.47796841053721
O	-4.00315736502075	-0.88651334498519	-0.75691512504175
H	-4.87627577501295	-0.87835957622961	-1.17080369750955
H	-3.34701657891256	-0.87144941112640	-1.50150048711821
O	-2.00089522952068	-0.65492498211350	-2.49531742792446
H	-1.47210837464148	-1.38627147256921	-2.86478286926319
H	-1.86801406586253	0.11021429567224	-3.08350947806432
O	1.14100864436650	-1.76148356712456	3.48501582823873
H	1.29726524944388	-2.01042294103750	4.40509559961933
H	0.16517006411795	-1.60732011327028	3.40299105589578
O	-0.20514374952158	-2.83600552555779	-2.83652923092191
H	-0.32912662519197	-3.68659567927923	-3.27833619001347
H	-0.37330427752563	-2.99059149957148	-1.87302352974320
O	-1.16279163370386	1.79155930161951	-3.68348597508024
H	-1.17232929787032	2.16576659238026	-4.57502891588917
H	-0.23092894288127	1.60018514730653	-3.47977872440830

O	1.95767167741954	-3.04169037414939	1.08363943179738
H	1.06904704286058	-3.09469900836221	0.67721662571995
H	1.77726753741794	-2.72389699941167	1.98955562857615
O	2.11784505730105	-1.31772582348570	-2.75612593096052
H	1.40885123514176	-1.98999155180570	-2.85851593517097
H	2.70247591873077	-1.62835192207935	-2.02930521013301
O	4.02123276009798	0.67991514415962	0.54404988245723
H	4.91261953212749	0.84787529206048	0.87863122667393
H	4.04808163061787	-0.22433275428065	0.15005881127205
O	3.67067218057197	-1.84375591896043	-0.50556247779808
H	4.41599796872983	-2.45036463951064	-0.61866453948377
H	3.05331163453440	-2.30696207128620	0.13770688147624

Lowest-energy isomer of Class XIII:

DLPNO-CCSD(T)/CBSest(zpe) energy relative to most stable Class I isomer: 1.34 kcal/mol

O	0.61245728082991	1.73722193214782	-1.75607397049052
H	0.26336980654573	0.99256636114193	-1.15761682520642
H	1.56118324463697	1.41777129942567	-2.07090827580461
H	0.69849288768599	2.54861535476405	-1.12422788326612
O	1.87173797939575	-1.59681756552323	-3.31405648845380
H	2.30294660382663	-2.22019036104063	-3.91347378994807
H	1.73819462090067	-2.07422527080305	-2.45933434269865
O	-2.58187334304310	-2.64303469621618	-1.68205133007813
H	-3.40315015778380	-2.96029128557321	-2.08125179969642
H	-2.84081021203652	-1.90773461677748	-1.07458508395091
O	-2.18838997377388	1.62597186655370	-3.26384406446911
H	-1.68428230692565	0.77933481015586	-3.24634004182323
H	-1.54175330984588	2.28685469159348	-3.54414214654870
O	-0.83943763942641	-0.71756987608624	-2.91183327232570
H	-0.01782066694063	-1.04146816672381	-3.32191491596705
H	-1.37329795561300	-1.50903156162836	-2.71904258903709
O	1.28177851535573	-2.36627134348663	-0.79031339142384
H	0.75315345209201	-1.57450983567731	-0.53432963845139
H	0.68627828405942	-3.11364839912238	-0.58123159628890
O	0.89664100573971	0.75964451690040	2.36762119747605
H	1.28219282412265	-0.01631280927433	2.83225059764709
H	1.62799803179996	1.39802287976099	2.24336865337939
O	-2.77262278796962	-0.54819111826153	0.00658326684330
H	-3.02822429733876	-0.66105645971236	0.95149492699423
H	-3.14710530837958	0.31404167216818	-0.28004162642620
O	-0.06995081310140	-0.06863178678025	-0.04524012815594
H	0.22705905743473	0.20670367697422	0.85813054171549
H	-1.03695534722052	-0.25909782706165	0.01367552700126
O	-0.85068442715612	-4.05403660540957	-0.06888028908758
H	-0.95309748301981	-5.01424986552449	-0.03175991736547
H	-1.54942771702103	-3.72979197487403	-0.68065071465864
O	-0.65677546287764	-2.49879944869083	2.32850839554833
H	-0.82888017559494	-3.08723726720165	1.57144457974640
H	-1.49211132549901	-2.03542354853478	2.52845793612394
O	1.69353952685306	-1.68854072342367	3.27484381232582
H	1.88582552505050	-2.03700971061244	4.15495342234079
H	0.83460156459794	-2.09992571999781	2.99787138571755
O	3.33388756329672	-1.59472975459972	0.95521702982152
H	2.69445736542275	-2.01797287321807	0.34650065387089
H	2.91639098927717	-1.69247012820882	1.83255733909727
O	4.12515637551004	0.72844689521446	0.01910539556986

H	5.09248588456293	0.71236021370934	0.00180424662184
H	3.85725043244557	-0.15311383911837	0.41878612230789
O	-2.92826960450115	-0.82868503162306	2.69885157915500
H	-3.64058964474020	-0.98347461987974	3.33288334382956
H	-2.53767428628481	0.06163738892521	2.92786893768622
O	2.79590146011213	2.61543661631193	1.52081639031816
H	3.38152899282741	3.16030307004743	2.06370497259338
H	3.38895431586024	2.02555327771519	0.99627983998902
O	2.84245969361144	0.85709837163415	-2.46452490366990
H	2.68332562222896	-0.03974934202831	-2.83217638546010
H	3.40667978216984	0.74318060116282	-1.66914341344938
O	-1.70504949118968	1.48796481194789	3.06230013037175
H	-0.74663640434328	1.31225264466749	2.97266925039627
H	-1.87427879794846	2.19411125268259	2.41176325447681
O	0.69884275706164	3.63574933483669	-0.08258528359243
H	1.39200214631314	3.43967684713795	0.57876139238266
H	-0.16298971316753	3.62633328398919	0.38428069311512
O	-3.39856739195175	1.97906645525462	-0.95302015836983
H	-4.30795572542841	2.24491080103505	-1.14851203659521
H	-2.95292621290429	1.90698062819297	-1.84816982173474
O	-1.93341823926244	3.40988110921290	0.91729232071830
H	-2.35334946035828	4.26116514879924	1.10383937225403
H	-2.49753690897975	2.99057661863064	0.22673661705890
